# Making sense of TILs: recommendations for morphological assessment of tumour‐infiltrating lymphocytes in gastro‐oesophageal carcinoma: A report on behalf of the International Immuno‐Oncology Biomarker Working Group

**DOI:** 10.1111/his.70089

**Published:** 2026-02-05

**Authors:** Ylva A Weeda, Roberto Salgado, Filip van Herpe, Daniel Sur, Michael Vieth, Sherene Loi, Ahmad P Tafti, Hardas Alexandros, Akira I Hida, Amarpreet Bhalla, Andrey I Khramtsov, Anna Ehinger, Atsushi Tanaka, Balazs Acs, Caner Ercan, Cornelia M Focke, Daniel Ehinger, Dan Huang, Galina F Khramtsova, Haruto Nishida, Nianyi Li, Nickolas Littlefield, Sanna Steen, Selim Sevim, Shin Ichihara, Shinnosuke Morikawa, Stefan Michiels, Thomas Papathomas, Toshihiro Haga, Qiangqiang Gu, Weiqi Sheng, Hoel Kervadec, Hugo M Horlings, Francisco Sanchez‐Vega, Yelena Y Janjigian, Myriam Chalabi, Hanneke W M van Laarhoven, Liudmila L Kodach, Sybren L Meijer

**Affiliations:** ^1^ Department of Pathology Amsterdam UMC Location University of Amsterdam Amsterdam The Netherlands; ^2^ Cancer Center Amsterdam Cancer Treatment and Quality of Life Amsterdam The Netherlands; ^3^ Department of Pathology ZAS Hospitals Brussels Antwerp Belgium; ^4^ Division of Cancer Research Peter MacCallum Cancer Centre Melbourne Victoria Australia; ^5^ GI Oncology, Department of Gastroenterology Catholic University Leuven, University Hospitals Leuven Leuven Belgium; ^6^ Department of Medical Oncology University of Medicine and Pharmacy “Iuliu Hațieganu” Cluj‐Napoca Romania; ^7^ Department of Medical Oncology The Oncology Institute “Prof. Dr. Ion Chiricuță” Cluj‐Napoca Romania; ^8^ Institute of Pathology, Klinikum Bayreuth Friedrich‐Alexander‐Universität Erlangen‐Nürnberg Bayreuth Germany; ^9^ The Sir Peter MacCallum Department of Oncology The University of Melbourne Parkville Victoria Australia; ^10^ Computational Pathology & AI Center of Excellence (CPACE), Department of Pathology University of Pittsburgh School of Medicine Pittsburgh Pennsylvania USA; ^11^ Intelligent Systems Program University of Pittsburgh Pittsburgh Pennsylvania USA; ^12^ School of Health and Rehabilitation Sciences University of Pittsburgh Pittsburgh Pennsylvania USA; ^13^ Department of Pathobiology & Population Sciences The Royal Veterinary College, University of London London UK; ^14^ Department of Pathology Matsuyama Shimin Hospital Matsuyama Japan; ^15^ Montefiore Medical Center The University Hospital for Albert Einstein College of Medicine Bronx New York USA; ^16^ Department of Pathology and Laboratory Medicine Ann & Robert H. Lurie Children's Hospital of Chicago Chicago Illinois USA; ^17^ Clinical Genetics, Pathology and Molecular Diagnostics Lund University Hospital Lund Sweden; ^18^ Department of Pathology Beth Israel Deaconess Medical Center, Harvard Medical School Boston Massachusetts USA; ^19^ Department of Clinical Pathology and Cancer Diagnostics Karolinska University Hospital Stockholm Sweden; ^20^ Department of Oncology‐Pathology Karolinska Institutet Stockholm Sweden; ^21^ Translational Molecular Pathology Department The University of Texas MD Anderson Cancer Center Houston Texas USA; ^22^ Department of Surgical Pathology Dietrich Bonhoeffer Klinikum Neubrandenburg Germany; ^23^ Department of Clinical Sciences Lund, Division of Oncology Lund University Lund Sweden; ^24^ Department of Genetics, Pathology, and Molecular Diagnostics Skåne University Hospital Helsingborg Sweden; ^25^ Department of Pathology Fudan University Shanghai Cancer Center Shanghai China; ^26^ Department of Oncology, Shanghai Medical College Fudan University Shanghai China; ^27^ Department of Medicine, Section of Hematology and Oncology, Center for Clinical Cancer Genetics and Global Health University of Chicago Chicago Illinois USA; ^28^ Department of Diagnostic Pathology, Faculty of Medicine Oita University Oita Japan; ^29^ Cancer Early Detection Advanced Research Center (CEDAR), Knight Cancer Institute Oregon Health and Science University Portland Oregon USA; ^30^ Department of Surgical Pathology Sapporo Kosei General Hospital Chuo‐Ku Japan; ^31^ Bureau de Biostatistique et d'Épidémiologie Gustave Roussy, Université Paris‐Saclay Villejuif France; ^32^ Oncostat, Inserm U1018, Équipe Labellisée Ligue Contre le Cancer Université Paris‐Saclay Villejuif France; ^33^ Department of Clinical Pathology Vestre Viken HF Drammen Norway; ^34^ University of Birmingham Institute of Metabolism and Systems Research Birmingham UK; ^35^ Department of Diagnostic Pathology National Cancer Center Hospital Tokyo Japan; ^36^ Department of Biomedical Engineering and Physics Amsterdam University Medical Centers Amsterdam The Netherlands; ^37^ Quantitative Healthcare Analysis Group, Informatics Institute University of Amsterdam Amsterdam The Netherlands; ^38^ Department of Pathology The Netherlands Cancer Institute Amsterdam The Netherlands; ^39^ Department of Epidemiology and Statistics Memorial Sloan Kettering, Computational Oncology Service New York New York USA; ^40^ Department of Surgery Memorial Sloan Kettering, Colorectal Service New York New York USA; ^41^ Department of Medicine Memorial Sloan Kettering Cancer Center New York New York USA; ^42^ Department of Medical Oncology The Netherlands Cancer Institute Amsterdam The Netherlands; ^43^ Department of Medical Oncology Amsterdam UMC Location University of Amsterdam Amsterdam The Netherlands

**Keywords:** gastro‐oesophageal cancer, tumour‐infiltrating lymphocytes

## Abstract

In the era of immune checkpoint inhibitors for cancers, the need for prognostic biomarkers to identify patients most likely to achieve a durable response has become increasingly more relevant. Tumour‐infiltrating lymphocytes (TILs) have gained significant interest, as they can be evaluated using standard haematoxylin and eosin‐stained slides, making it a widely accessible and cost‐effective biomarker. In addition to their practicality, TILs provide prognostic insights into the interplay between the immune system and tumour cells. While the morphological assessment of TILs has been standardised in breast cancer, comprehensive guidelines for their evaluation in gastro‐oesophageal carcinomas (GEC) are still lacking. This narrative review examines the current literature on the composition, clinical implications and therapeutic utility of TILs in GEC. These insights are used to propose a framework with recommendations for standardised evaluation and reporting of TILs in GEC, while also highlighting pitfalls specific to GEC pathology. These recommendations serve as a vital first step towards the widespread use and validation of TILs as a biomarker.

## Graphical abstract

This review examines the role of tumour‐infiltrating lymphocytes (TILs) in gastro‐oesophageal carcinoma (GEC), focusing on their composition, clinical relevance and therapeutic utility. It proposes a framework for standardised evaluation and reporting of TILs, addressing GEC‐specific pathology challenges to support the widespread use and validation of TILs as a predictive biomarker.

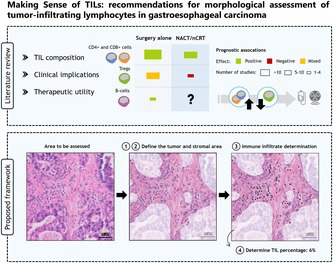

**Abbreviations**
EA: oesophageal adenocarcinomas
EFS: event‐free survival
ESCC: oesophageal squamous cell carcinoma
GA: gastric adenocarcinomas
GEA: gastric and oesophageal adenocarcinoma
GEC: gastro‐oesophageal carcinomas
H&E: haematoxylin and eosin
ICI: immune checkpoint inhibitors
IHC: immunohistochemistry
IM: immune‐modulated
IS: immune‐suppressed
ITWG: International Immuno‐Oncology Biomarker Working Group
NACT: neo‐adjuvant chemotherapy
nCRT: neo‐adjuvant chemoradiation
OS: overall survival
PD‐1: programmed‐death‐1
PD‐L1: programmed‐death‐ligand 1
TIL: tumour‐infiltrating lymphocytes
TME: tumour microenvironment

## Background

Gastro‐oesophageal cancer (GEC) accounts for 13% of cancer‐related deaths worldwide.^1^ Prognosis in patients with advanced or metastatic GEC who are ineligible for targeted therapies is remarkably poor, with a median overall survival of less than 12 months.^2^ Recently immune checkpoint inhibitors (ICIs) specific to the programmed‐death‐1 (PD‐1) receptor and its ligand programmed‐death‐ligand 1 (PD‐L1) have been incorporated into the treatment of various solid tumours. The European Medicines Agency approved several ICIs for GEC patients, including nivolumab,^3^, ^4^ pembrolizumab^5^, ^6^ and tislelizumab.^7^ These treatments have been incorporated into both international and national guidelines for gastric and oesophageal cancer management.^8^, ^9^ Although ICIs hold great potential with favourable long‐term outcomes, durable response is restricted to only a subset of patients.^10^, ^11^ With novel clinical indications on the rise,^12^, ^13^ appropriate patient selection is warranted to avoid side effects and healthcare costs in futile treatment.

In GEC patients, PD‐L1 expression is commonly used to guide ICI treatment decisions.^8^, ^9^ A significant drawback of this prognostic biomarker is that it requires immunohistochemistry (IHC) analysis, a process that is labour‐intensive. As a result, accessibility to this method may be limited in certain settings. In addition, PD‐L1 assessment is further complicated by high interobserver variability^14^ and the use of multiple scoring methods with inconsistent thresholds, including combined positive score (≥1, ≥5 or ≥10), tumour positive score (≥1) and tumour area positivity (≥0%, ≥1%, ≥5% or ≥10%).^15^ There is ongoing debate about whether tumours with low PD‐L1 expression may still benefit from immunotherapy, and whether a more accurate predictor of response is needed^16^, ^17^.

In the search for optimal biomarkers, tumour‐infiltrating lymphocytes (TILs) have gained increasing attention as key immune components within the tumour microenvironment (TME). Unlike IHC‐based biomarkers, TILs can be assessed through standard haematoxylin and eosin (H&E) staining, making their evaluation more accessible and less expensive, while also providing insights into tumour–immune interactions. TIL density and spatial distribution have proven to be strong indicators for treatment efficacy and resistance across various cancer types, particularly in breast cancer and melanoma.^18^, ^19^, ^20^ The International Immuno‐Oncology Biomarker Working Group (ITWG) established recommendations for standardised morphological assessment of TILs for breast cancer.^21^ However, comprehensive recommendations for TIL assessment in GEC do not exist yet. To uncover the possible potential of TILs as a predictive biomarker for ICI therapy in GEC and to enable widespread application of TILs as a biomarker in both research and clinical practice, it is essential to standardise evaluation and reporting of TILs in GEC.

The aim of this narrative review is, therefore, to summarise the current literature on the composition, clinical implications and therapeutic utility of TILs in GEC. Based on these insights, we outline a structured framework to guide pathologists in TIL assessment, addressing the specific challenges and histopathological characteristics of GEC. This framework aims to provide practical guidance to facilitate and standardise TIL assessment in daily practice.

## TIL Composition and Clinical Implications

Gastric adenocarcinomas (GA) and oesophageal adenocarcinomas (EAs) exhibit a wide range of baseline lymphocytic infiltration, which varies among molecular subtypes. For instance, mismatch repair‐deficient tumours^22^ and Epstein–Barr virus‐associated GAs^23^, ^24^, ^25^ are characterised by elevated levels of immune infiltration. The Lauren classification categorised GA into three subtypes based on histological characteristics: intestinal, diffuse and mixed‐type tumours. The intestinal phenotype^26^, ^27^ tends to exhibit moderate to high baseline immune infiltration, whereas the diffuse type^28^ is generally considered immune ‘cold’ with minimal cytotoxic T‐cell infiltration and low PD‐L1 expression, which is associated with more aggressive tumour behaviour. It has even been demonstrated that the extent of immune infiltration can differ per geographical location, with non‐Asian GAs showing significantly higher numbers of CD3+, CD4+ and CD8+ T cells, compared to Asian GAs.^29^ In oesophageal squamous cell carcinoma (ESCC), immune‐suppressed (IS) and immune‐modulated (IM) subtypes have been identified, both characterised by increased immune infiltration. The IM subtype is enriched in CD8+ T cells and macrophages, whereas the IS subtype shows a distinct immune composition with higher proportions of B cells and natural killer cells.^30^ Geographical differences in immune infiltration appear less prominent in ESCC than in GA.^31^

The presence of an inflamed TME, particularly one rich in CD8+ cytotoxic T cells, has been shown to have prognostic significance. A high number of CD8+ cytotoxic T cells, in resection specimens from GEC patients treated with surgery alone, has generally been associated with favourable survival outcomes.^32^, ^33^, ^34^, ^35^, ^36^, ^37^, ^38^, ^39^, ^40^, ^41^, ^42^ CD8+ cytotoxic T cells are the most prevalent subset of TILs; however, various alternative CD8+ T‐cell subsets with distinct functional characteristics, such as memory^43^ and exhausted T cells,^44^, ^45^ have been identified across different cancer types. Some of these subsets contribute more effectively to tumour elimination than others, and an imbalance in these subsets may influence prognosis. Nonetheless, in GEC, research into the differentiation of these CD8+ T‐cell subsets is still in its early stages. The role of FOXP3+ regulatory T cells in GEC patients treated with surgery alone remains controversial, with high densities being linked to both poor and favourable prognoses.^33^, ^35^, ^36^, ^46^, ^47^, ^48^, ^49^, ^50^, ^51^ Multiple studies even reported an association between high FOXP3+ cell densities post‐surgery and improved survival metrics,^33^, ^35^, ^36^, ^47^, ^51^ despite the fact that FOXP3+ regulatory T cells are primarily described as immunosuppressive. This seemingly paradoxical observation has also been observed in colorectal cancer patients, which was attributed to the phenotypical and functional heterogeneity of regulatory T cells.^52^, ^53^, ^54^ Two distinct subpopulations with immunosuppressive (effector Tregs) and non‐suppressive (naïve Tregs) characteristics can be identified, potentially explaining varying clinical outcomes.^47^, ^52^ Another possible explanation can be the fact that the increase in FOXP3+ cells may simply reflect an overall increase in T lymphocytes (CD4+ and CD8+).^51^ In some studies, neither FOXP3+, CD8+ nor CD4+ T cells were significantly related to patient prognosis. However, when these lymphocyte subsets were combined as ratios (e.g. high CD4+/CD8+ or CD8+/FOXP3+ ratios and low FOXP3+/CD4+ ratios), they emerged as independent favourable prognostic factors.^55^, ^56^, ^57^

Nowadays treatment of locally advanced GECs includes multimodality regimens, consisting of neo‐adjuvant chemotherapy (NACT) or neo‐adjuvant chemoradiation (nCRT), which influences the composition of the immune landscape. Markedly higher numbers of CD4+ and CD8+ T cells^58^, ^59^, ^60^, ^61^ along with reduced numbers of FOXP3+ T cells^62^ were observed in tumours of resected GEC patients who received preoperative treatment compared to patients who were treated with surgery alone. Similarly, when comparing pretreatment biopsies and post‐treatment specimens in GEC patients, an influx of CD4+ and CD8+ T cells was observed in post‐treatment samples,^62^, ^63^, ^64^, ^65^, ^66^, ^67^, ^68^ while the regulatory FOXP3+ T‐cell subset tended to reduce over the course of NACT and nCRT regimens.^63^, ^65^, ^69^ This difference highlights the ability of preoperative treatments to reshape the TME. Interestingly, elevated levels of CD4+ and CD8+ cells prior to treatment initiation (nCRT and chemo immunotherapy regimens) were associated with prolonged survival in ESCC^70^ patients, suggesting that the composition of immune infiltrate in pretreatment biopsies could potentially serve as a predictive marker for treatment efficacy. A high number of T cells post‐treatment is generally associated with histopathological evidence of tumour regression,^70^, ^71^, ^72^ radiological response^66^ and favourable prognosis.^65^, ^70^, ^71^, ^72^ However, high densities of CD8+ T cells following preoperative treatment can also be observed in poor responders, which may reflect a dysfunctional immune response, where infiltrating cytotoxic T cells fail to effectively contribute to tumour elimination.^73^ Low densities of FOXP3+ T cells post‐treatment were associated with favourable prognoses in GEC.^63^, ^68^, ^74^

The increase in T‐cell infiltration induced by NACT or nCRT can create a synergistic effect when combined with ICIs, as these therapies work most effectively in an immune‐activated environment. Studies examining the changes in the immune landscape of GEC patients receiving ICI regimens are limited. However, higher levels of CD8+ T cells have been reported in pathological responders to ICIs, both as monotherapy and in combination with chemotherapy, in patients with locally advanced EA^12^ and advanced GA^75^. In ESCC patients, tissue‐resident memory T cells (CD103+ CD8+) have gained interest as a potential immunotherapeutic target,^76^ as they have been associated with prolonged survival in advanced ESCC patients treated with combined chemotherapy and ICI^77^. There is convincing data that chemotherapy alone primes the TME to evolve in the direction of immunogenic cell death, thereby enhancing susceptibility to immunotherapy. In metastatic GA patients with radiological response to ICIs, expansion of naïve, memory and exhausted CD8+ T cells was observed after treatment initiation, whereas this was not the case in slow responders.^45^ These CD8+ T cells represent a subset of the TIL population, reinforcing the need to better understand how the TME evolves in response to combined treatment strategies.

The TIL population also includes B cells, which can make up a significant portion of the TILs. For instance, in breast cancer, they account for up to 40% of the total TIL population.^78^ The prognostic value of infiltrating B cells and plasma cells in gastro‐oesophageal cancer remains elusive, and is primarily studied in conjunction with peritumosural tertiary lymphoid structures (TLS).^79^, ^80^, ^81^ Studies in gastric and oesophageal adenocarcinoma (GEA) patients undergoing surgery have demonstrated that elevated post‐surgical levels of CD20+ and CD138+ cells are associated with prolonged survival.^80^, ^82^ However, studies investigating the impact of B cells over the course of neo‐adjuvant treatment remain limited. Insights from other tumour types suggest that B cells play a significant role, as high levels post‐treatment are often associated with favourable survival outcomes.^83^, ^84^, ^85^ Nonetheless, plasma cells are commonly found in the healthy gastrointestinal tract due to the continuous exposure to microorganisms.^86^ Moreover, as a result, it remains uncertain to what extent B cells and plasma cells contribute to tumour cell elimination or if they simply reflect non‐specific resident inflammation.

**Figure 1:**
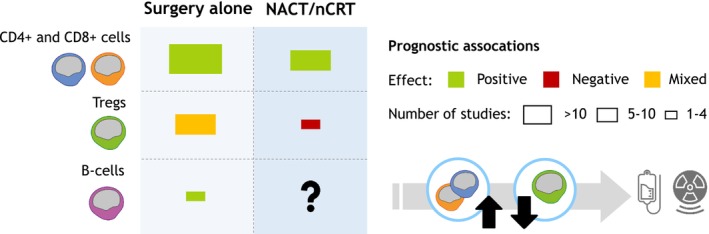
Schematic overview to summarise the literature on TIL composition and clinical implications. The way of quantifying the prognostic associations was adapted from Laumont et al. Over the course of neo‐adjuvant chemotherapy or chemoradiation, CD4± and CD8± T cells are increased, while regulatory T cells decrease. NACT, neo‐adjuvant chemotherapy; nCRT, neo‐adjuvant chemoradiation; Tregs, regulatory T cells.

In conclusion, the composition of TILs in GEC cancers holds significant prognostic value. Some tumour subtypes naturally exhibit higher levels of immune infiltration at baseline. Furthermore, NACT and nCRT reshape the immune landscape towards a more immune stimulating environment. This shift involves an increase in T‐helper cells (CD4+) and cytotoxic T cells (CD8+) while reducing regulatory T cells (FOXP3+). The prognostic associations of these immune cell types vary depending on the treatment approach, which is visualised in Figure 1.^87^

## Therapeutic Utility of Morphological TIL Evaluation

The previous paragraph outlined the composition of TILs and the various immune cell subsets involved. In clinical practice, TILs are assessed morphologically on H&E‐stained slides as a single entity, without distinguishing between specific immune cell types. In the early stages of morphological TIL evaluation in H&E sections of GEC patients, TIL density was often reported using simplified categorical values (e.g. absent, moderate, high).^88^, ^89^, ^90^ More standardised methods were also reported, which were guidelines adopted from colorectal cancer. For instance, the Klintrup criteria^91^ were used to assess the inflammatory infiltrate at the tumour‐invasive margin.^92^, ^93^ The system categorises TIL density in four groups from absent or minimal inflammation to more prominent, destructive infiltrates that form distinct patterns, including ‘cup‐like’ structures. The Ropponen classification system^94^ combines both the TIL quantity (0, 1–30, 30–50 and >50 cells per field) and density (none, weak, moderate and dense) to create four categories.^95^, ^96^ Due to varying methodological approaches, comparing these scoring methods is challenging.

The ITWG recommendations for breast cancer defined TILs as a semiquantitative measure expressed as a percentage, reflecting the proportion of the area occupied by mononuclear infiltrate (i.e. lymphocytes and plasma cells). TILs can be evaluated in distinct tumour regions: stromal TILs are assessed within the tumour stroma, while intratumoural TILs are scored within the tumour bulk, where TILs are in direct contact with cancer cells. The term ‘intratumoural TILs’ is, however, used in the literature with varying definitions, sometimes referring exclusively to the tumour cell compartment, while in other contexts encompassing both the stromal and tumour cell compartments. Intratumoural TILs were hypothesised to hold biological significance, as they are in closer proximity to cancer cells.^21^ In studies evaluating both compartments in oesophageal cancer patients, stromal TILs generally retained their prognostic value in multivariate analyses, whereas intratumoural TILs lost their significance.^97^, ^98^, ^99^ This may be attributed to the fact that intratumoural TILs in these specimens were found to be extremely low and difficult to assess.^98^

Current standard of care for localised GEC includes nCRT followed by adjuvant ICI treatment,^100^ perioperative chemotherapy^101^, ^102^ or definitive CRT^103^, ^104^ A shift is now occurring with the addition of ICI therapy in localised disease. Keynote‐585 evaluated the addition of pembrolizumab to chemotherapy, showing a numerical improvement in event‐free survival (EFS) compared to patients treated with chemotherapy alone, but did not meet the primary endpoint of overall survival (OS).^105^ While higher PD‐L1 CPS scores were associated with improved pathological complete response, it did not translate into a survival benefit. The MATTERHORN trial demonstrated a significant EFS benefit from adding durvalumab to perioperative chemotherapy, although no significant OS benefit was observed.^106^, ^107^ Notably, this study used the PD‐L1 TAP score for patient stratification, rather than CPS. PD‐L1 CPS, TPS and TAP scores are currently driving clinical decision‐making. However, given their inconsistencies, there is a growing need for more robust biomarkers to predict immunotherapy response. TILs have emerged as a promising and accessible alternative.

High stromal TILs in resection specimens from GEC patients, whether treated with surgery alone or NACT, were consistently associated with prolonged survival across multiple studies.^97^, ^99^, ^108^, ^109^, ^110^, ^111^, ^112^, ^113^ Patients with high baseline TIL density were more likely to experience both radiological response to nCRT and prolonged survival compared to those with low TIL density,^98^, ^114^ underscoring the predictive value of TILs. A similar trend was observed in oesophageal cancer specimens collected prior to ICI treatment,^115^ highlighting their potential role in the selection of patients likely to respond to immunotherapy. Due to the limited sample size, definitive conclusions on the added value of TILs as a predictive biomarker for immunotherapy response are limited. In the future, we would therefore recommend a retrospective analysis to investigate the added value of TILs and compare whether TILs outperform PD‐L1 CPS, TPS or TAP scores. Second, if TILs are shown to be predictive of immunotherapy response, this could enable identification of true responders in GEC and support the exploration of novel drug opportunities for patients with low‐TIL tumors.

The question remains: what defines a clinically relevant TIL‐high score in GEC patients? Reported cut‐offs varied widely, ranging from 10% to 50%, depending on the patient population studied. For instance, relatively higher TIL levels have been observed in EBV‐associated gastric cancers.^108^, ^109^ However, in studies involving ESCC patients, a 50% cut‐off was not often reported, as only a few patients exhibited TIL percentages that high.^97^, ^114^ Cut‐offs mentioned in articles are derived from specific patient populations and cannot be reliably extrapolated to broader real‐world settings, as none have been validated on external datasets. In retrospective research, it is feasible to investigate novel thresholds for the predictive value of TILs in GEC. Given this premature research, it is recommended to initially assess TIL density as a continuous variable. This approach allows for identification of potential cut‐off values that could guide dichotomization strategies in future prospective trials, ultimately supporting the validation of TILs as a clinically relevant biomarker.

In conclusion, stromal TILs are preferred for morphological evaluation of GEC specimens, as they consistently demonstrate prognostic value and serve as a reliable biomarker for survival outcomes. Although PD‐L1 scoring is currently used for (immune)‐response stratification, the variability in scoring systems and cut‐off thresholds limits its robustness. TILs represent a promising alternative and should be compared to the existing CPS, TPS and TAP in future studies. In breast cancer, the current recommendation by the Working Group is to evaluate both TILs as well as PD‐L1.^116^ A similar development may be envisaged in GEC as well as in other tumor types. To establish a clinically relevant threshold for high TIL density, it is essential to assess TILs as a continuous variable, preserving prognostic detail and avoiding arbitrary dichotomization in the absence of validated cut‐off values.

## Methodological Recommendations for Evaluating TILs in GEC

**Figure 2:**
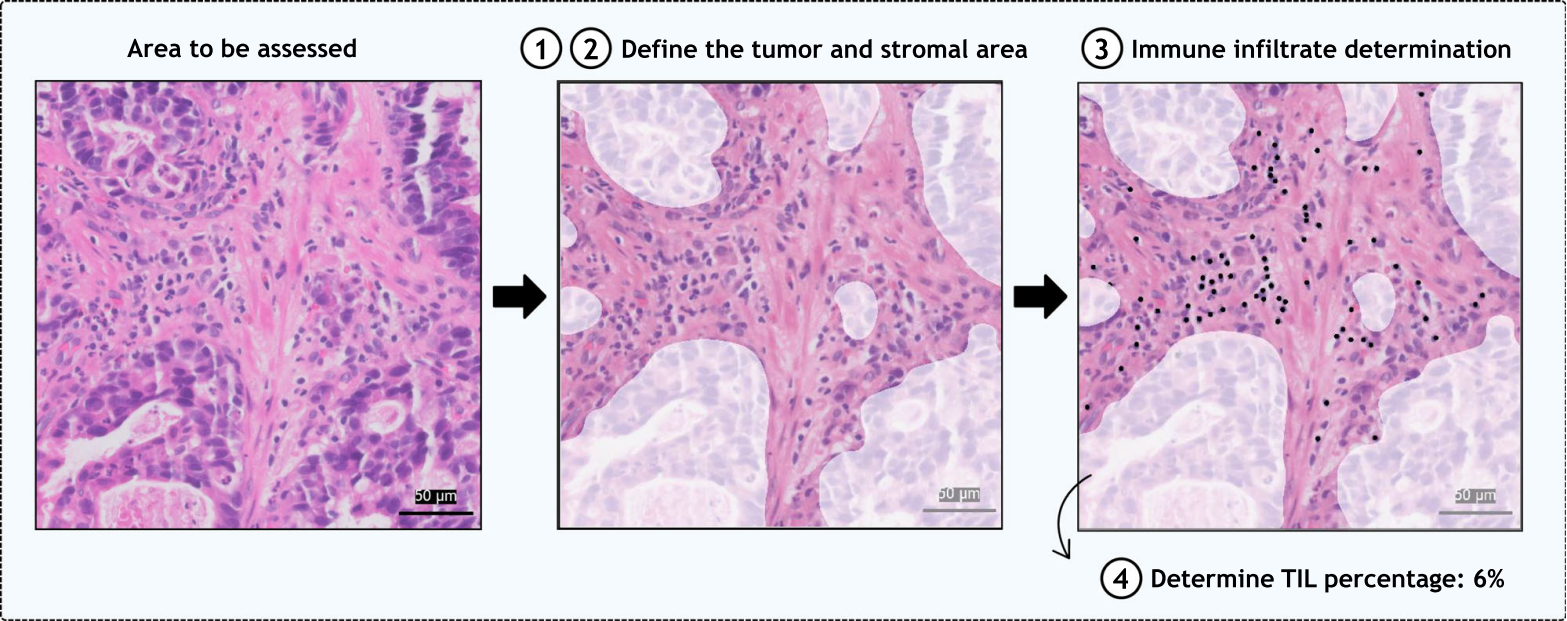
Proposed framework for TIL assessment in gastro‐oesophageal carcinoma specimens, following these key steps: (1) define the tumour area, (2) define the stromal area, (3) immune infiltrate determination and (4) determine the TIL percentage. The steps are illustrated using a selected region from a whole slide image of a patient with oesophageal adenocarcinoma.

Standardisation of TIL scoring is an important step towards establishing the predictive value of this biomarker, clinically relevant cut‐offs for immunotherapy response and potential use in daily clinical practice. It is also essential to use comparable approaches and consistent steps in TIL assessment across different tumour types. Based on the current literature, we have developed a structured framework for the evaluation of TILs in GEC. Importantly, this guideline for GEC is consistent with the ITWG recommendations for TIL evaluation in other tumour types. This framework is intended to facilitate TIL evaluation by providing a standardised approach to TIL scoring rather than to dictate treatment decisions in GEC patients. Currently, there is insufficient evidence to determine cut‐offs for high or low TILs. Cut‐offs may vary depending on tumour subtypes and whether assessments are made pre‐ or post‐treatment. Further research, including internal and external validation, is therefore essential to establish accurate and reliable thresholds. This guideline should therefore be applied thoughtfully, considering the specific clinical or research context in which it is implemented. The framework follows four steps: (i) Define tumour area, (ii) define stromal area, (iii) determine the type of immune infiltrate and (iv) determine the percentage of TILs (Figure 2). A concise tutorial following the above‐mentioned steps was developed (Data S1), along with a summary Table S1, and can be found on the ITWG website (https://www.tilsinbreastcancer.org/).

### Define Tumor Area

**Figure 3:**
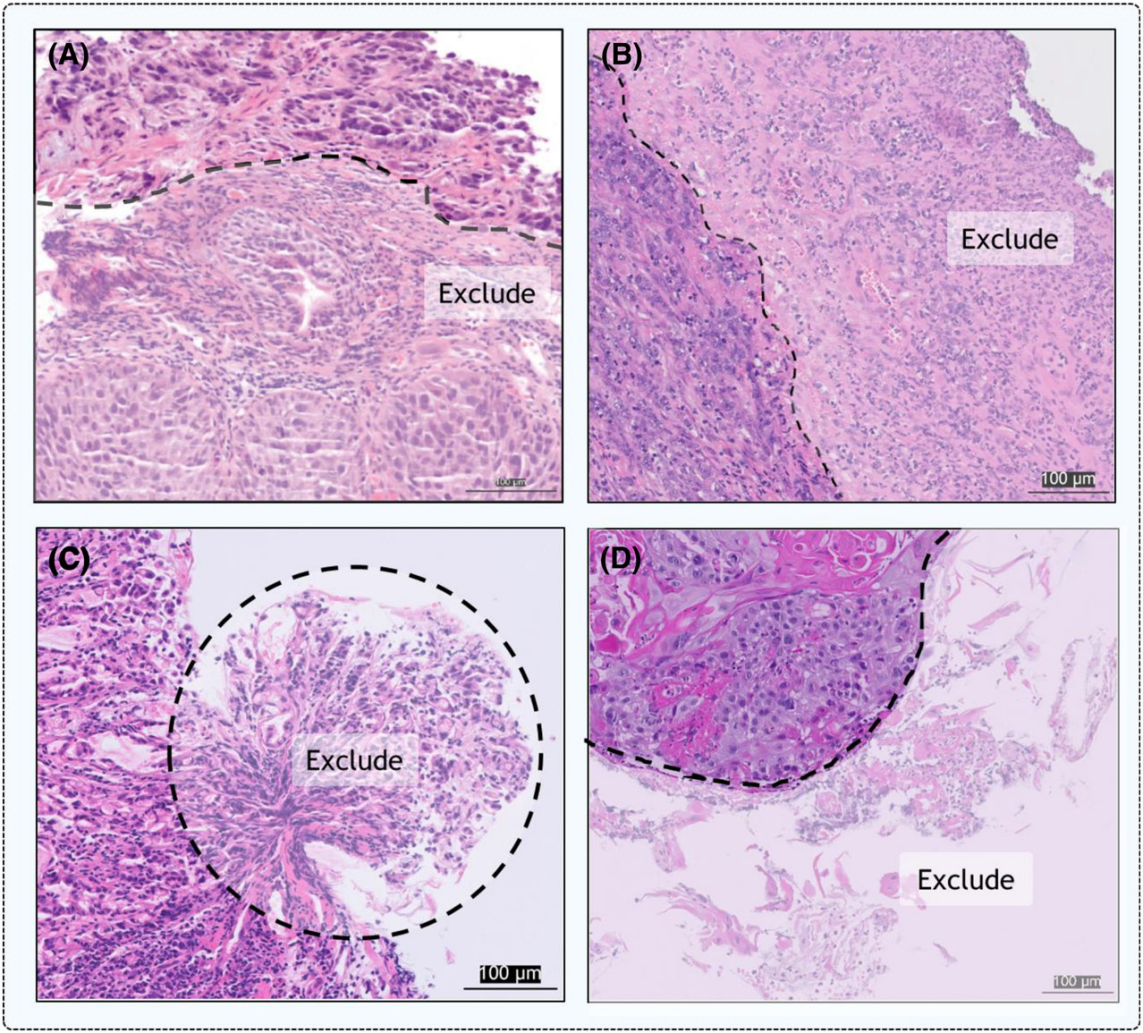
Examples of regions that should be excluded from scoring: (**A**) areas of dysplasia, (**B**) superficial ulceration, (**C**) pinch artefacts and (**D**) loose fragments. The latter image was normalised for visual consistency. All scale bars are equal to 100 microns. [Colour figure can be viewed at wileyonlinelibrary.com]

TILs should be assessed strictly within the borders of the invasive tumour. Immune cells located outside the tumour border, including those within tertiary lymphoid structures (TLS), areas of dysplasia (Figure 3a), normal glands or non‐malignant mucosa, should be excluded. Similarly, large areas of necrosis, superficial erosions, ulcerations (Figure 3b), debris, crush artefacts (Figure 3c) and loose fragments (Figure 3d) should not be considered.

### Define Stromal Area

**Figure 4:**
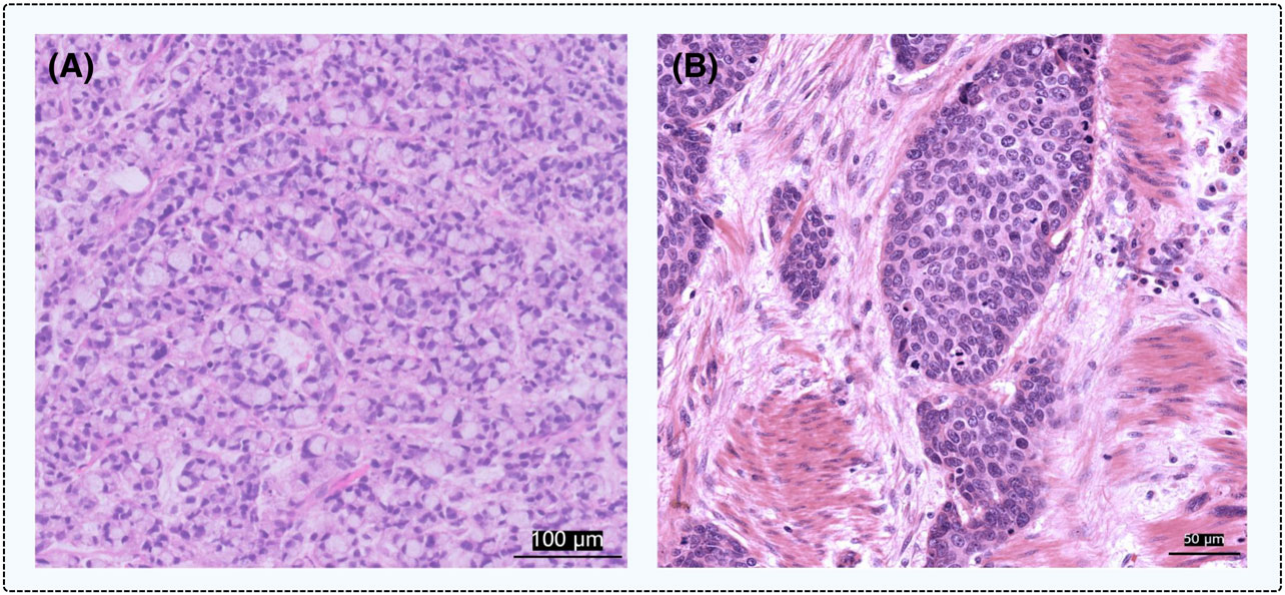
Examples of regions where defining the stromal area can be challenging: (**A**) signet ring cell carcinoma of the stomach with minimal stromal content (<10%). (**B**) Invasive growth of oesophageal squamous cell carcinoma within muscle tissue. The image was normalised for visual consistency. The corresponding TIL percentages for these areas are both <5%. Scale bars are equal to 100 and 50 microns. [Colour figure can be viewed at wileyonlinelibrary.com]

Stromal TILs have demonstrated prognostic value and are more commonly used in research than intratumoural TILs, likely due to the challenges associated with assessing intratumoural TILs in GEC. To enhance reproducibility, we therefore recommend focusing solely on the stromal compartment of the tumour bulk. Defining tumour‐associated stroma can sometimes be challenging, especially in poorly cohesive adenocarcinomas and tumours with low stromal content (Figure 4a). When tumour and stroma cannot be reliably distinguished, TILs should be reported as a single total score without distinguishing between compartments. Distinguishing between muscle and stromal tissue complicates scoring, as pathologists must not only filter out tumour areas but also visually distinguish and exclude muscle tissue, adding an extra step to the entire process (Figure 4b). We recommend to exclude thick muscle fibres of the muscularis mucosae and muscularis propria, while thin solitary muscle fibres intermingling with collagen fibres can be included. Additionally, extracellular mucin, intraluminal space of malignant glands and intratumoural blood vessels should not be evaluated.

### Determine the Type of Immune Infiltrate

**Figure 5:**
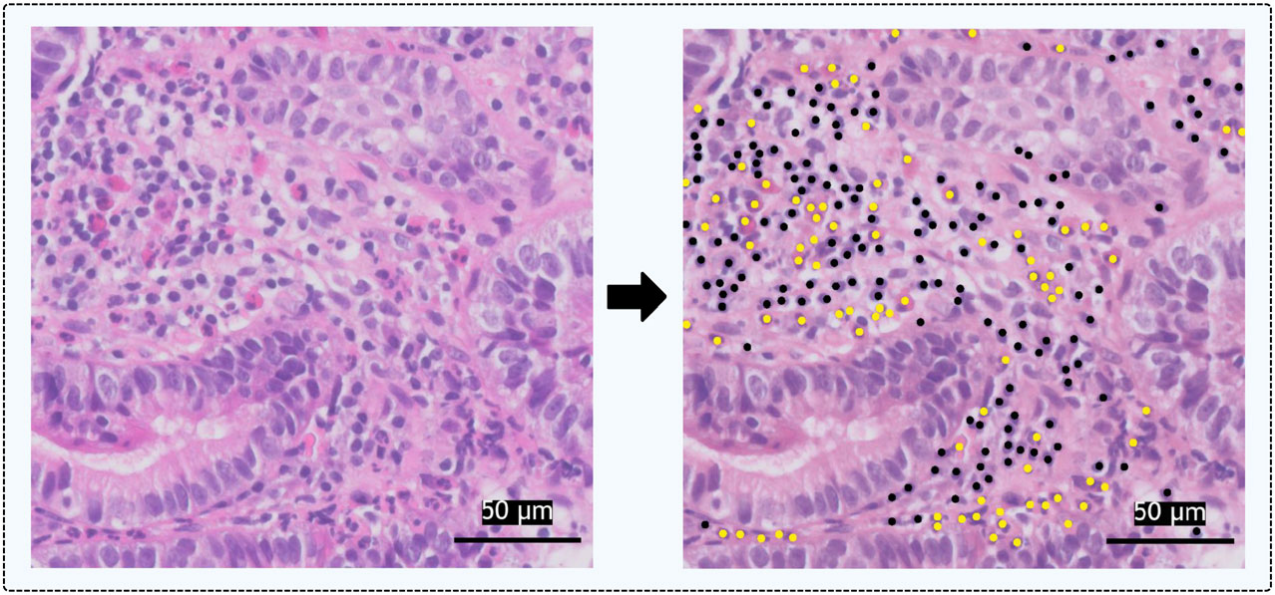
Example of a region from an oesophageal adenocarcinoma sample, where TILs (black dots) and granulocytes (yellow dots) are manually annotated. Granulocytes make up approximately 35% of the total immune infiltrate in this area. Granulocytes and other polymorphonuclear cells are frequently found in gastro‐oesophageal malignancies, but should be excluded from the final TIL score. The scale bar equals 50 microns. [Colour figure can be viewed at wileyonlinelibrary.com]

All mononuclear cells, including lymphocytes and plasma cells, should be scored. The prognostic value of plasma cells alone is not yet established in GEC. However, distinguishing plasma cells from other immune cells based on morphology is challenging, making their exclusion from the score impractical. A similar approach is used in the ITWG recommendations on the assessment of TILs in breast cancer, where plasma cells are included in the TIL score to maintain consistency and simplicity. Following this precedent, we recommend incorporating plasma cells in the scoring process for GEC as well. Granulocytes and other polymorphonuclear cells, though abundant in GEC tissue, should not be included in the final TIL score (Figure 5).

### Determine the Percentage of Stromal TILs

**Figure 6:**
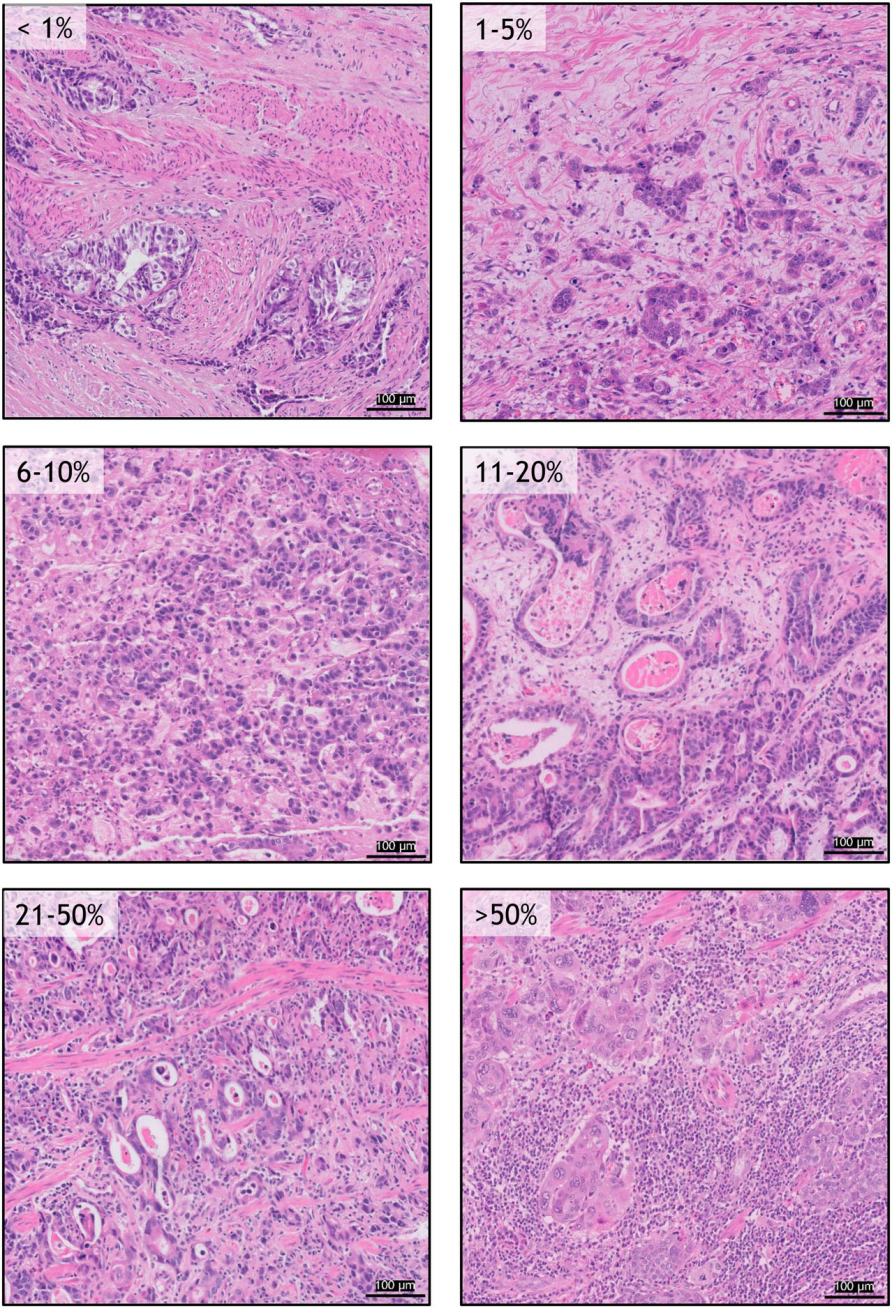
Reference images of gastro‐oesophageal adenocarcinomas and oesophageal squamous cell carcinomas divided into six categories (<1%, 1%–5%, 6%–10%, 11%–20%, 21%–50% and >50%). All images were obtained using a 0.5‐mm^2^ bounding box and were normalised for visual consistency. All scale bars are equal to 100 microns. [Colour figure can be viewed at wileyonlinelibrary.com]

When determining the percentage of stromal TILs, it is important to avoid focusing solely on hotspots. This is particularly relevant in heterogeneous tumours, such as GEAs, where spatial heterogeneity (e.g. in HER2 expression) is more pronounced than in breast cancer.^117^, ^118^ Averaging multiple areas of interest, therefore, provides a more representative score.^119^ To further support TIL assessment in GEC, reference images were created for six defined groups (<1%, 1%–5%, 6%–10%, 11%–20%, 21%–50% and >50%) (Figure 6). These images serve as a visual guide to enhance scoring consistency. Using a lower magnification can be helpful when evaluating intermediate categories (11%–50%). Pathologists should report their scores as a continuous variable, providing as much detail as they deem appropriate. The use of semi‐quantitative scoring systems likely reduces interpathologist variability, as humans tend to agree more easily when working within clearly defined groups. Nonetheless, for robust statistical analyses and reliable inter‐study comparisons, continuous variables are preferred.

## Pitfalls

**Figure 7:**
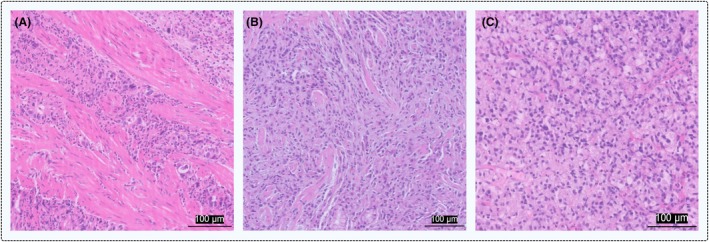
Examples of pitfalls. (**A**) Poorly cohesive adenocarcinoma with high cell density (>70%), where the immune infiltrate is largely composed of neutrophils, which may lead to an overestimation of the TIL score. The actual TIL percentage is only 5%. (**B**) Poorly cohesive adenocarcinoma where distinguishing between the tumour and stromal compartments is challenging. In such cases, the TIL percentage should be assessed across the entire area without separation, yielding a score of 10%. (**C**) Signet ring cell adenocarcinoma in which TILs and tumour cells appear morphologically similar, complicating accurate assessment. The TIL score in this area was reported between 5% and 10%. Zooming out to obtain a global view can aid differentiation. All scale bars are equal to 100 microns. [Colour figure can be viewed at wileyonlinelibrary.com]

While the full range of pitfalls has not yet been identified, there are some specific issues that are known to cause problems when evaluating TILs. In certain complex cases, multiple histopathological characteristics overlap, such as an abundant presence of granulocytes, muscle fibres and limited stromal area. Regions with high cell density can be particularly challenging to assess, especially when a large proportion of the immune infiltrate is composed of neutrophils (Figure 7a). Additionally, distinguishing stroma from tumour tissue can be difficult in some areas and the TIL percentage should be assessed across the entire area without separation between the two compartments (Figure 7b). Small tumour cells may also resemble TILs, complicating accurate scoring and potentially leading to overestimation (Figure 7c). In cases where tumour cells and TILs appear morphologically similar, zooming out to assess overall patterns can aid differentiation.

## Future Prospects

The framework outlined in this study provides a foundation for the standardised evaluation and reporting of TILs in GEC. As part of this framework, decision support tools incorporating integrated visual reference feedback can be developed and implemented to enhance consistency in TIL scoring and reduce interobserver variability among pathologists.^120^ One of the initial steps in establishing TILs as a robust predictive biomarker is to implement widespread TIL scoring and assess its therapeutic utility in retrospective studies, correlating TIL scores with clinical outcomes. Moving from retrospective to prospective studies will be essential for establishing the usefulness of TILs as a predictive biomarker for therapy response and for defining reliable clinically relevant TIL cut‐off values.

Improving current predictive biomarkers for immunotherapy response remains essential, as the various PD‐L1 scoring systems (CPS, TPS and TAP) continue to cause clinical ambiguity. With emerging indications of immunotherapy integration into the treatment of localised disease, it is important to define responders to justify the added value of immunotherapy. Equally important is the early identification of non‐responders, enabling alternative treatment strategies. In this context, we aim to validate TILs as a novel predictive biomarker in the future.

Furthermore, deep learning approaches hold great promise for TIL evaluation in GEC,^121^, ^122^, ^123^, ^124^ as the vast size of whole slide images makes visual scoring challenging for pathologists. These methods allow for quantitative outcome measures for TIL densities (cells/mm^2^). They also enable analysis of spatial characteristics, including clustering patterns and the proximity of TILs to tumour cells. Nonetheless, current GEC studies typically assess TILs across the entire tissue section without distinguishing between tumour‐associated stroma and the intraepithelial compartment. Future studies should address this limitation and explore the feasibility of identifying specific TIL subgroups, such as plasma cells, to assess their individual prognostic value.

## Author contributions

Conceptualization: Y.A.W., L.L.K., S.L.M. and R.S. Methodology: Y.A.W., L.L.K., S.L.M. and R.S. Investigation: Y.A.W. Writing—original draft: Y.A.W., F.v.H., L.L.K, S.L.M. Writing—review & editing: Y.A.W., R.S., F.v.H., D.S., M.V., S.L., A.P.T., H.A., A.I.H., A.B., A.I.K., A.E., A.T., B.A., C.E., C.M.F., D.E., D.H., G.F.K., H.N., N.L., N.Li., S.S., S.Sev., S.I., S.M., S.Mi., T.P., T.H., Q.G., W.S., H.K., H.M.H., F.S.‐V., Y.Y.J., M.C., H.W.M.v.L., L.L.K. and S.L.M. Supervision: L.L.K., S.L.M. and R.S. Funding Acquisition: S.L.M.

## Funding information

Y.A.W. was supported by a public grant from the ‘Koningin Wilhelmina Fonds’ (KWF150591). The funder had no role in the design of the study, in the collection, analyses or interpretation of data, in the writing of the manuscript or in the decision to publish the results.

## Conflict of interests

Roberto Salgado: Advisory Board role for BMS, Roche, Exact Sciences, Daicchii Sankyo, Astra Zeneca. Research funding by Roche, Puma, Merck, BMS and Travel and congress‐registration support by Roche, Merck, Astra Zeneca. Filip van Herpe: Grants/Research Support: Astellas, Amgen, Roche diagnostics, Speakers Bureau/Honoraria: MSD, Consulting Fees: Need Inc., Amgen, Travel grants: AstraZeneca, Servier, Merck, Ipsen, Roche. Balazs Acs: supported by The Swedish Society for Medical Research (Svenska Sällskapet för Medicinsk Forskning) postdoctoral grant and by Region Stockholm (clinical research appointment). Personal fees for consultancy to Pfizer. Stefan Michiels: fees for Scientific Committee Study member: Roche, for Data and safety monitoring member of clinical trials: IQVIA, Kedrion, Servier, Yuhan. Yelena Y Janjigian: Research funding: Astellas, AstraZeneca, Arcus Biosciences, Bayer, Bristol‐Myers Squibb, Cycle for Survival, Department of Defense, Eli Lilly, Fred's Team, Genentech/Roche, Inspirna, Merck, NCI, Stand Up 2 Cancer, Transcenta. Advisory boards/consulting: Abbvie, Alphasights, Amerisource Bergen, Ask‐Gene Pharma, Inc., Arcus Biosciences, Astellas, Astra Zeneca, Basilea Pharmaceutica, Bayer, BeOne, Boehringer Ingelheim, Bristol‐Myers Squibb, Clinical Care Options, Daiichi‐Sankyo, Debbie's Dream Foundation, eChina Health, Ed Med Resources (OncInfo), Eisai, Eli Lilly, Geneos Therapeutics, Gilead Sciences, GlaxoSmithKline, Guardant Health, Inc., H.C. Wainwright & Co., Health, Advances, HMP Global, I3Health, Imedex, Imugene, Inspirna, Lynx Health, Mashup Media LLC, Master Clinician Alliance, Merck, Merck Serono, Mersana, Therapeutics, Michael J. Hennessy Associates, Oncology News, OncoDaily, Paradigm Medical, Communications, PeerMD, PeerView Institute, Pfizer, Physician's Education Resource, LLC, Research to Practice, Sanofi Genzyme, Seagen, Silverback, Therapeutics, Suzhou Liangyihui Network Technology, Co., Ltd, Talem Health, TotalCME, WebMD, Zymeworks Inc. Other: Inspirna(stock options), Veda Life Sciences, Inc. (stock options). Myriam Chalabi: advisor to Bristol Myers Squibb, Merck Sharp & Dohme, Pfizer and Roche/Genentech and has received research grants unrelated to this study from Bristol Myers Squibb, Agenus, Merck Sharp & Dohme and Roche/Genentech. All grants were paid to the institution. Hanneke W M van Laarhoven: Research funding and/or medication/material supply from: AMGEN, Auristone, BMS, Incyte, Merck, MyeloidTx, ORCA, Servier, Consultant/advisory role: AMGEN, Amphera, Astellas, AstraZeneca, Beigene, BMS, Boehringer, Daiichy, MSD, MyeloidTx, Speaker role: AstraZeneca, BMS, Congress Care, Daiichy, Medtalks, Uitgeverij JAAP, Travel Congress Management. The rest of the authors have declared no conflicts of interest. We affirm that this research has been conducted with integrity and in accordance with all ethical principles.

## Supporting information


**Data S1.** Upper_GI_tutorial.

**Table S1.** Proposed framework for TIL assessment in gastro‐oesophageal carcinoma specimens

## Data Availability

The data generated during/and or analysed during the current study are available from the corresponding authors upon reasonable request.
