# Supplementary material for: Making sense of TILs: recommendations for morphological assessment of tumour‐infiltrating lymphocytes in gastro‐oesophageal carcinoma: A report on behalf of the International Immuno‐Oncology Biomarker Working Group
Source: Histopathology. 2026 Feb 5;88(6):1126–41. doi: 10.1111/his.70089 (PMC13051458; doi:10.1111/his.70089)

Standardized approach for TIL evaluation in gastroesophageal carcinomas

Authors: Ylva A. Weeda[1,2] , Liudmila L. Kodach[3] & Sybren L. Meijer[1,2]
[1] Amsterdam UMC Location University of Amsterdam, Department of Pathology, Amsterdam, The Netherlands
[2] Cancer Center Amsterdam, Cancer Treatment and Quality of Life, Amsterdam, The Netherlands
[3] The Netherlands Cancer Institute, Department of Pathology, Amsterdam, The Netherlands

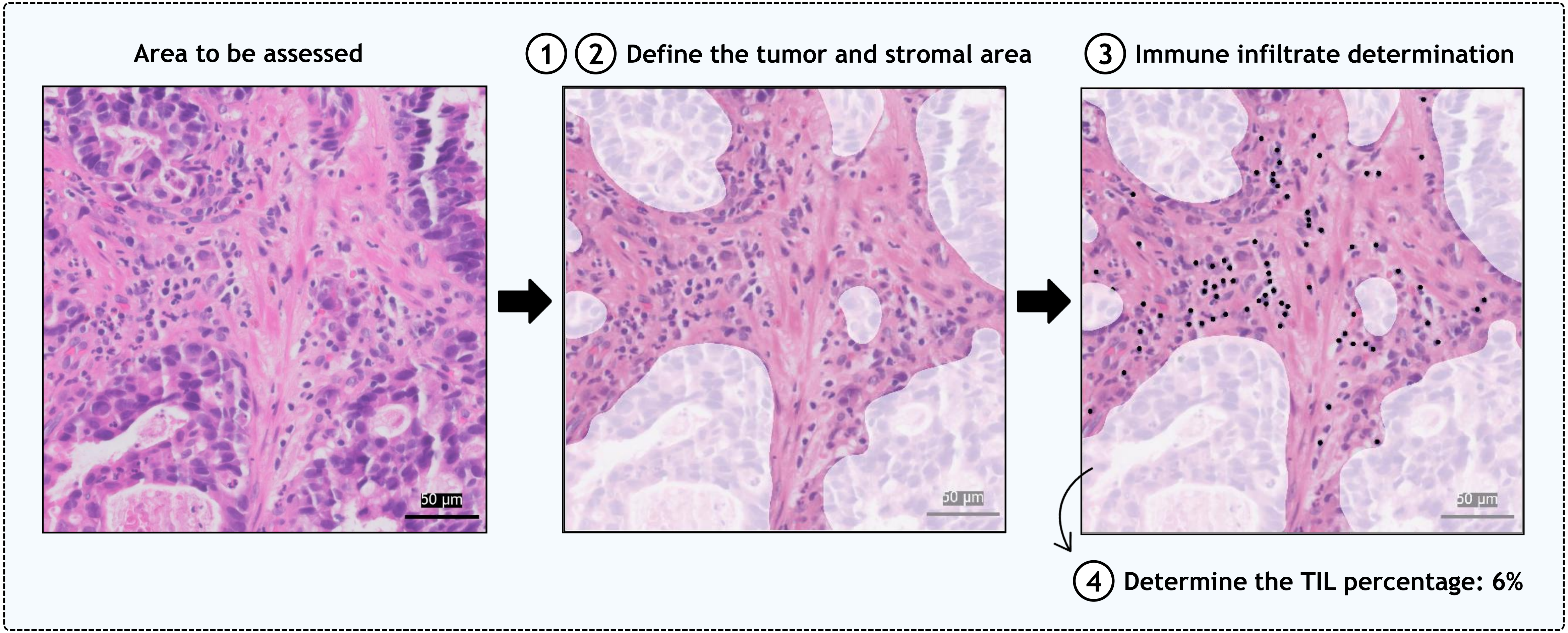
Area to be assessed
1 2 Define the tumor and stromal area
3 Immune infiltrate determination
50 µm
50 µm
50 µm
4 Determine the TIL percentage: 6%

Standardized approach for TIL evaluation in gastroesophageal carcinomas
**1. Define the tumor area**
TILs should be assessed within the borders of the invasive tumor. Exclude immune cells outside the tumor (e.g. tertiary lymphoid structures (TLS), areas of dysplasia, normal glands, or non-malignant mucosa). Exclude areas with superficial erosions, ulcerations, debris, crush artifacts and loose fragments.
**2. Define the stromal area**
Focus exclusively on stromal TILs. Exclude thick muscle fibers from the muscularis mucosae and muscularis propria. Thin muscle fibers intermingling with collagen may be included. Exclude extracellular mucin, intraluminal space of malignant glands and intratumoral blood vessels. If tumor and stroma are indistinguishable, report TILs as a single total score without compartment distinction.
**3. Determine the type of immune infiltrate**
All mononuclear cells, including lymphocytes and plasma cells, should be scored. Avoid the inclusion of granulocytes and other polymorphonuclear cells.
**4. Determine the percentage of stromal TILs**
Report the average of TILs in the stromal area, do not focus on hotspots. For intermediate groups (11-50%) evaluate different areas at higher magnification. Pathologists should report their scores as a continuous variable, providing as much detail as they deem appropriate. For robust statistical analyses and reliable inter-study comparisons, continuous variables are preferred.

Step 1. Define the tumor area

- TILs should be assessed within the borders of the invasive tumor

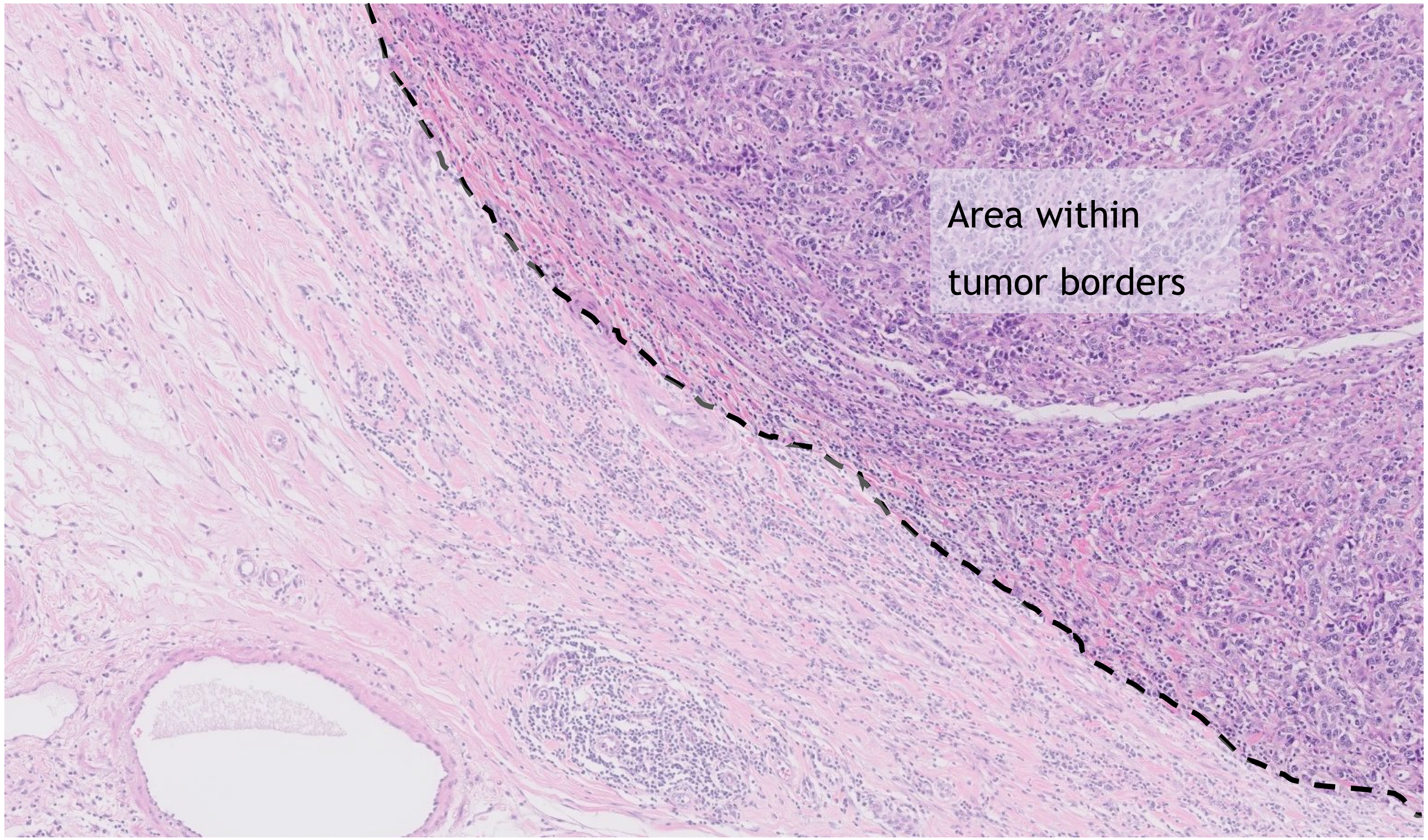

Step 1. Define the tumor area

- TILs should be assessed within the borders of the invasive tumor
- Exclude immune cells outside of the tumor borders (e.g. **tertiary lymphoid structures (TLS)**, areas of dysplasia, normal glands or non-malignant mucosa)

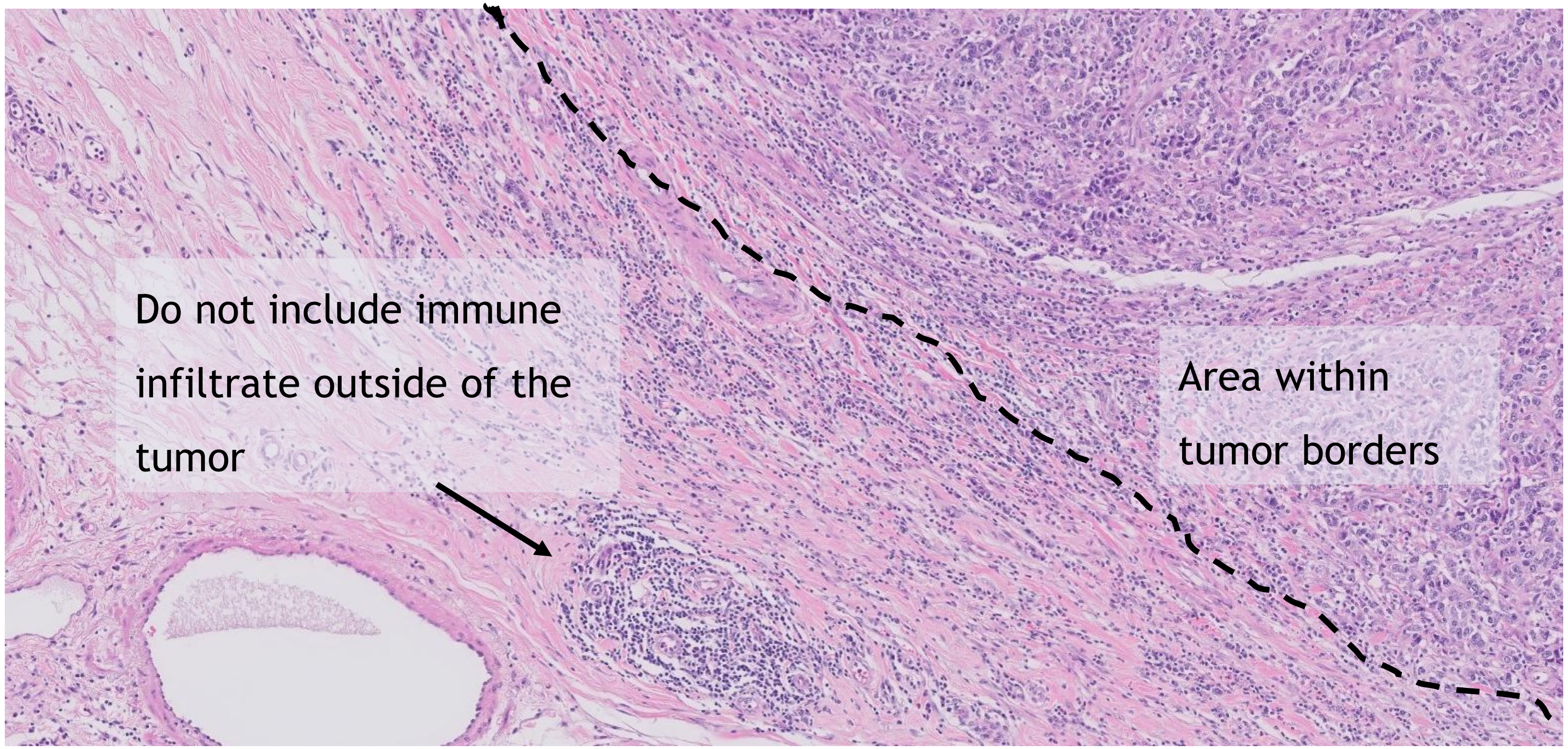

Step 1. Define the tumor area

- TILs should be assessed within the borders of the invasive tumor
- Exclude areas with necrosis, superficial erosions, **ulcerations**, debris, crush artifacts and loose fragments should not be considered

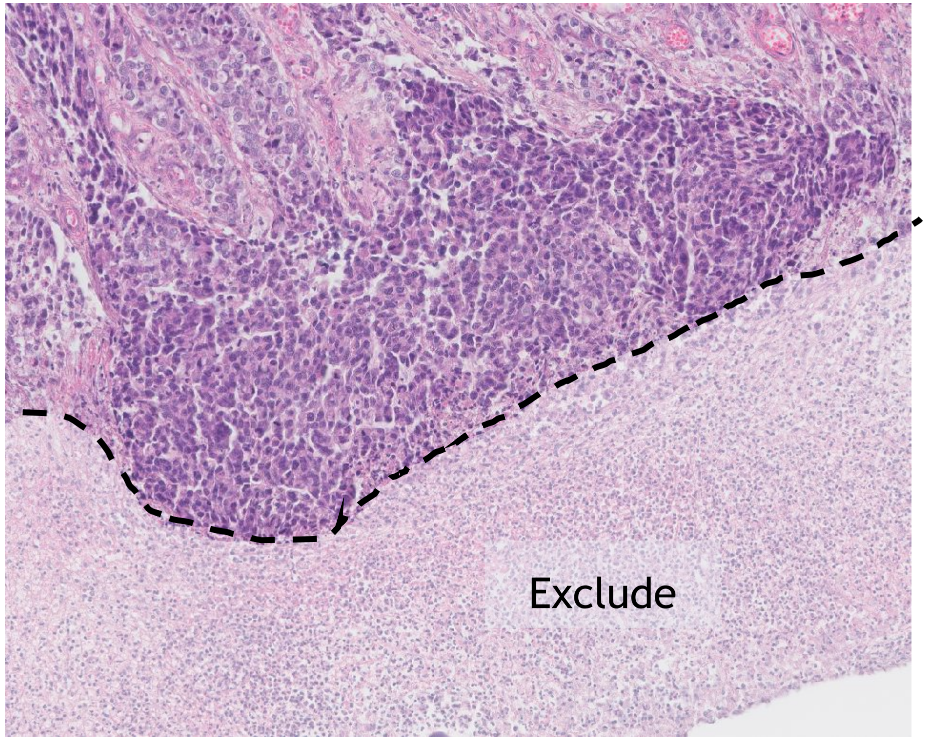

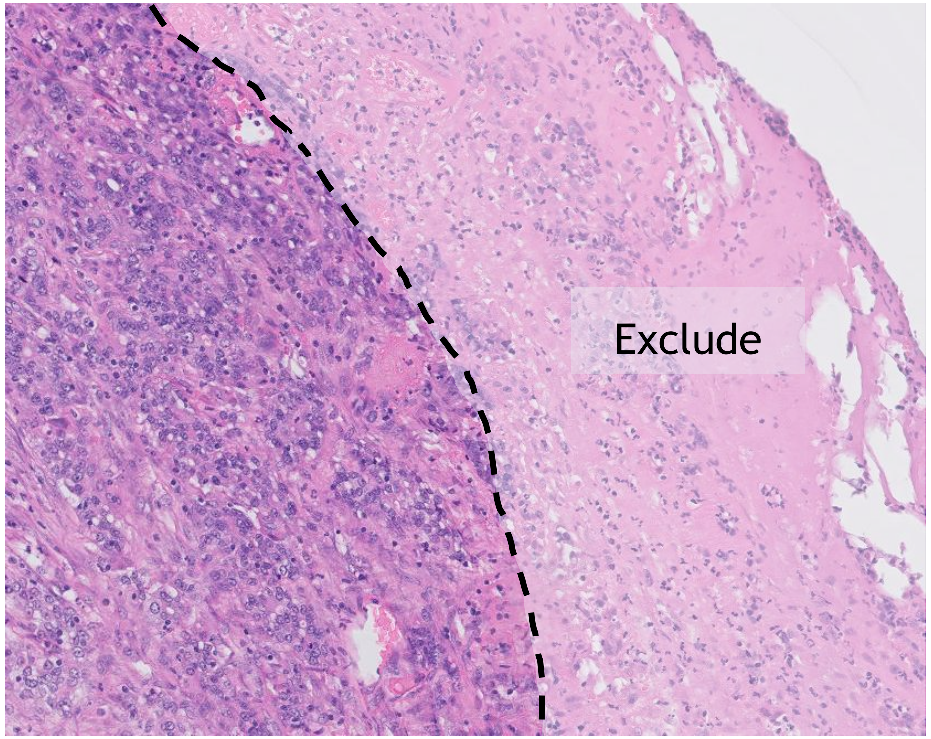

Step 1. Define the tumor area

- TILs should be assessed within the borders of the invasive tumor
- Exclude areas with necrosis, superficial erosions, ulcerations, debris, **crush artifacts** and loose fragments should not be considered

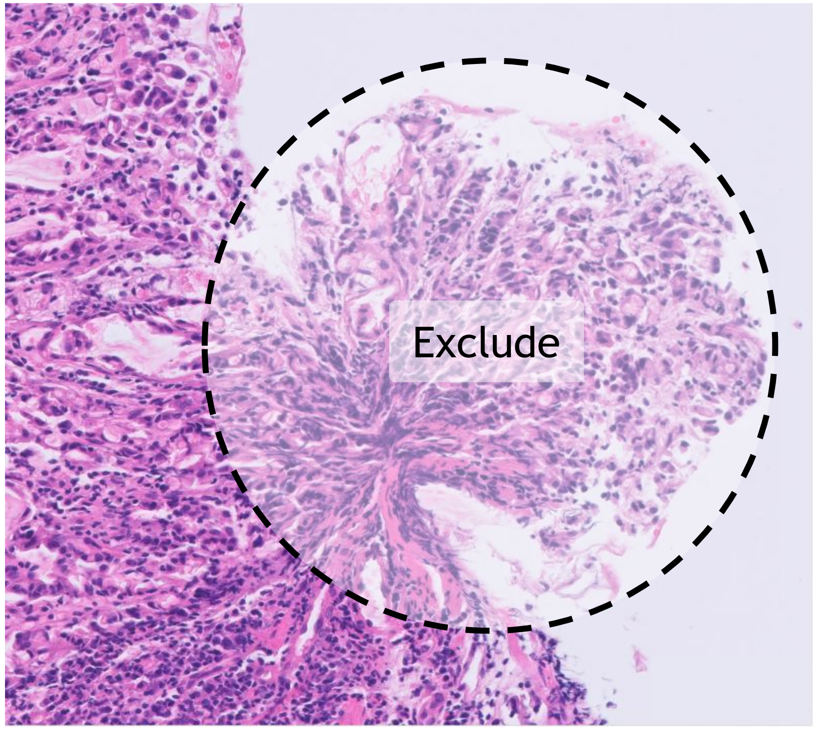

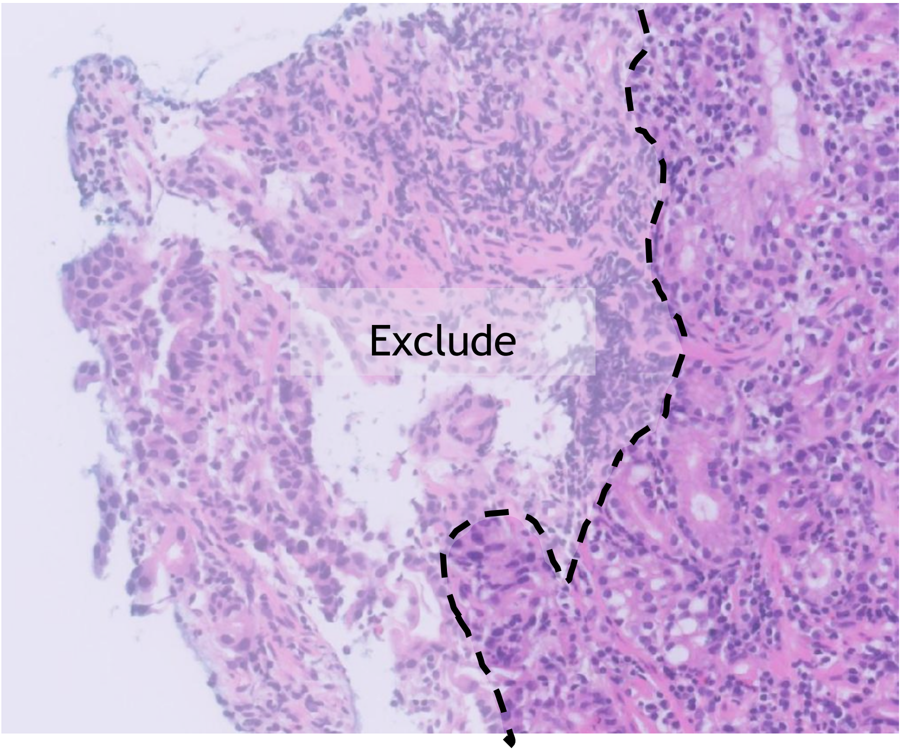

Step 1. Define the tumor area

- TILs should be assessed within the borders of the invasive tumor
- Exclude areas with necrosis, superficial erosions, ulcerations, debris, crush artifacts and **loose fragments** should not be considered

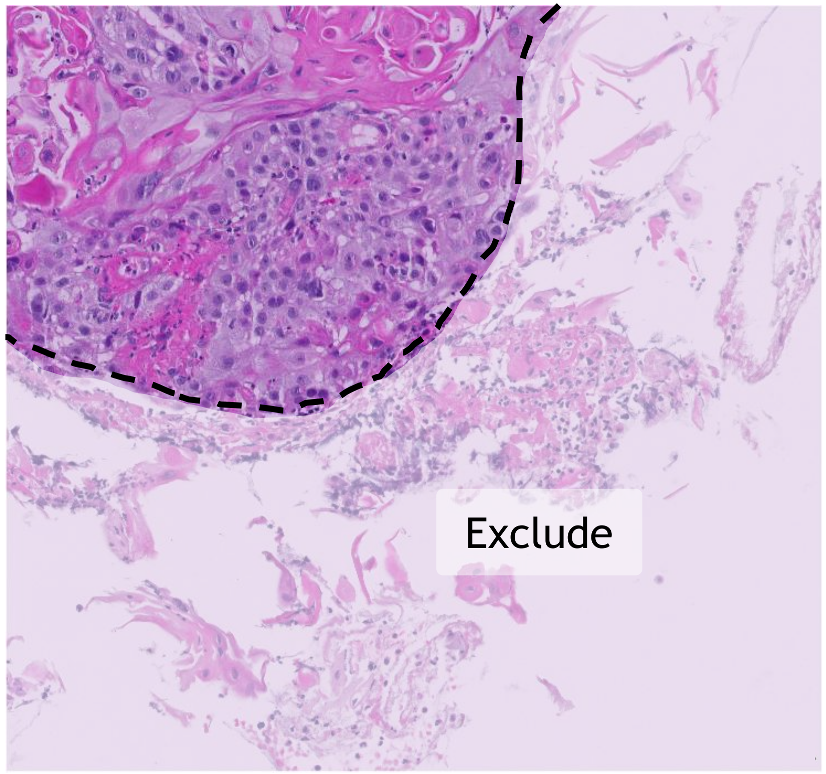

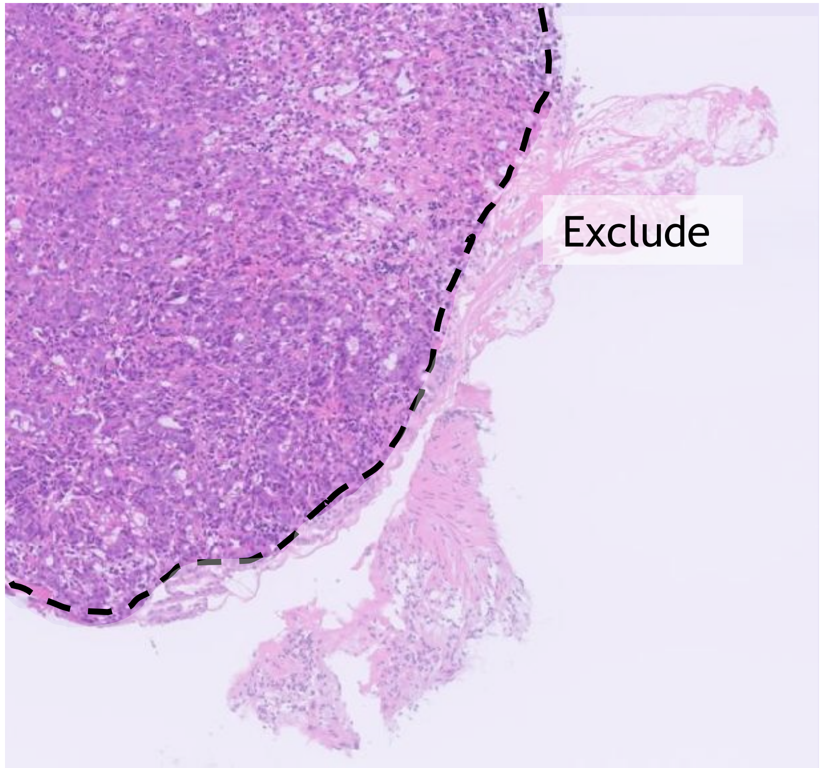

Step 2. Define the stromal area

- Focus exclusively on stromal TILs

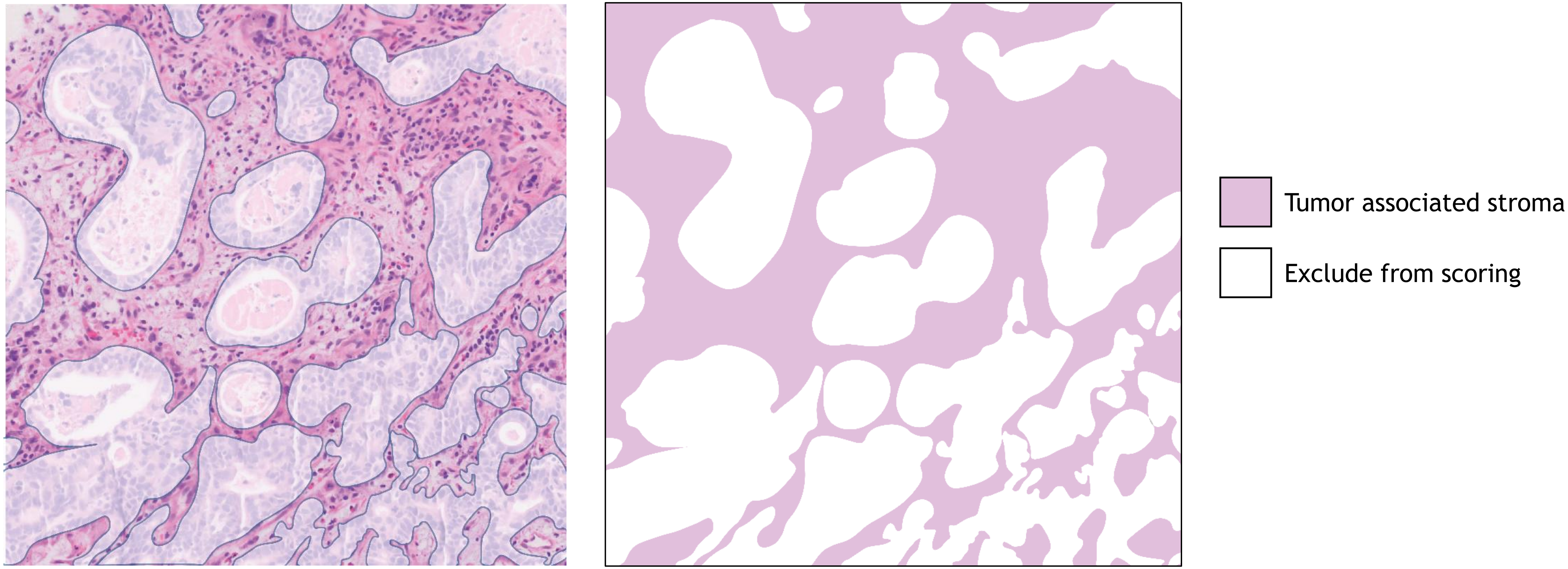

Step 2. Define the stromal area

- Focus exclusively on stromal TILs
- Exclude thick muscle fibers of the m. mucosae and m. propria

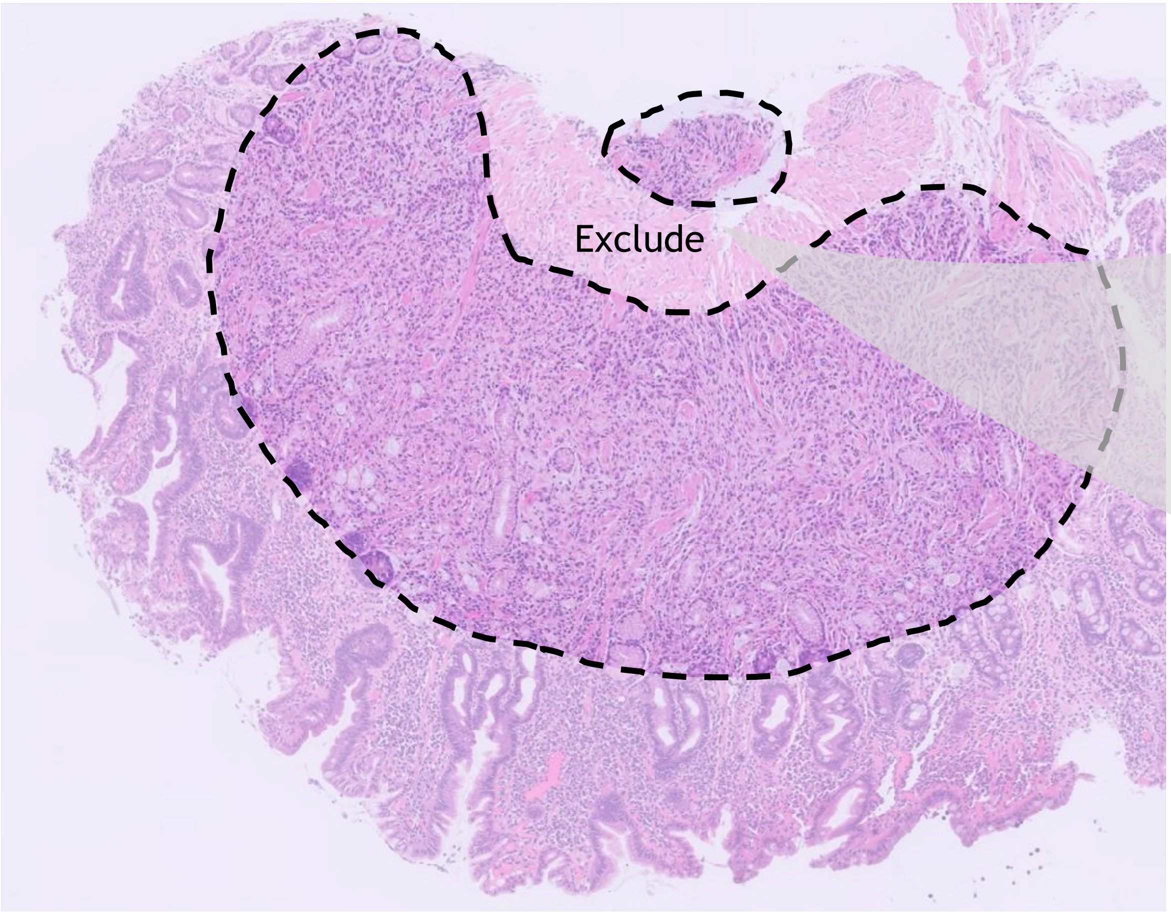

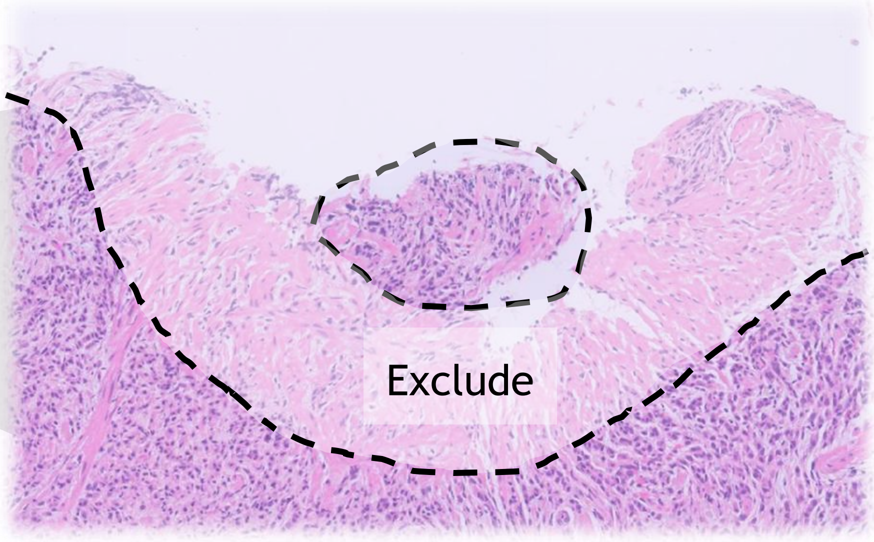

Step 2. Define the stromal area

- Focus exclusively on stromal TILs
- Exclude thick muscle fibers of the m. mucosae and m. propria

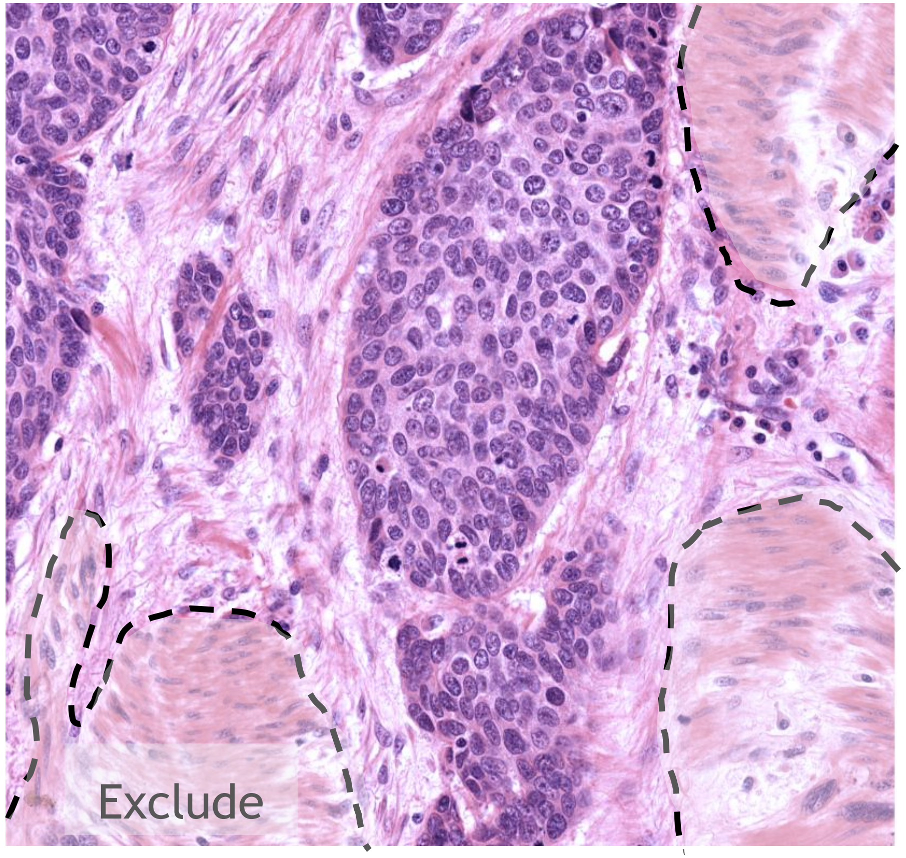

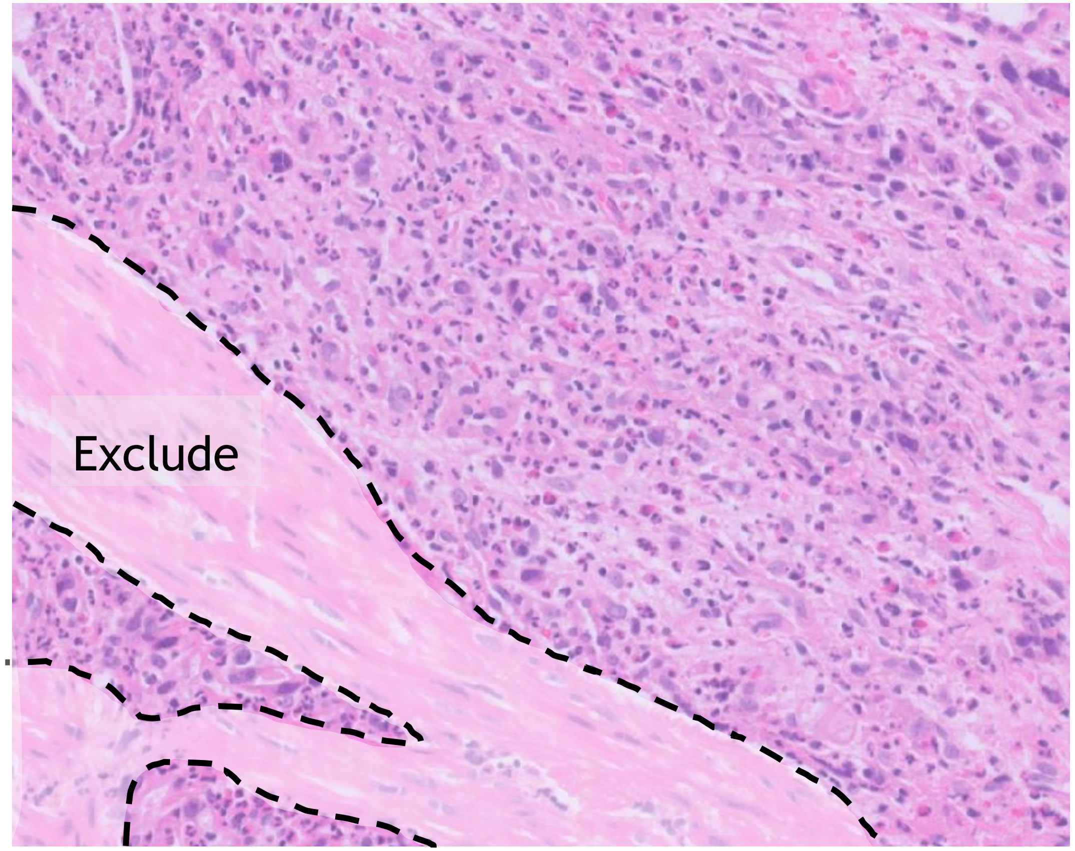

Step 2. Define the stromal area

- Focus exclusively on stromal TILs
- Thin solitary muscle intermingling with collagen fibers can be included

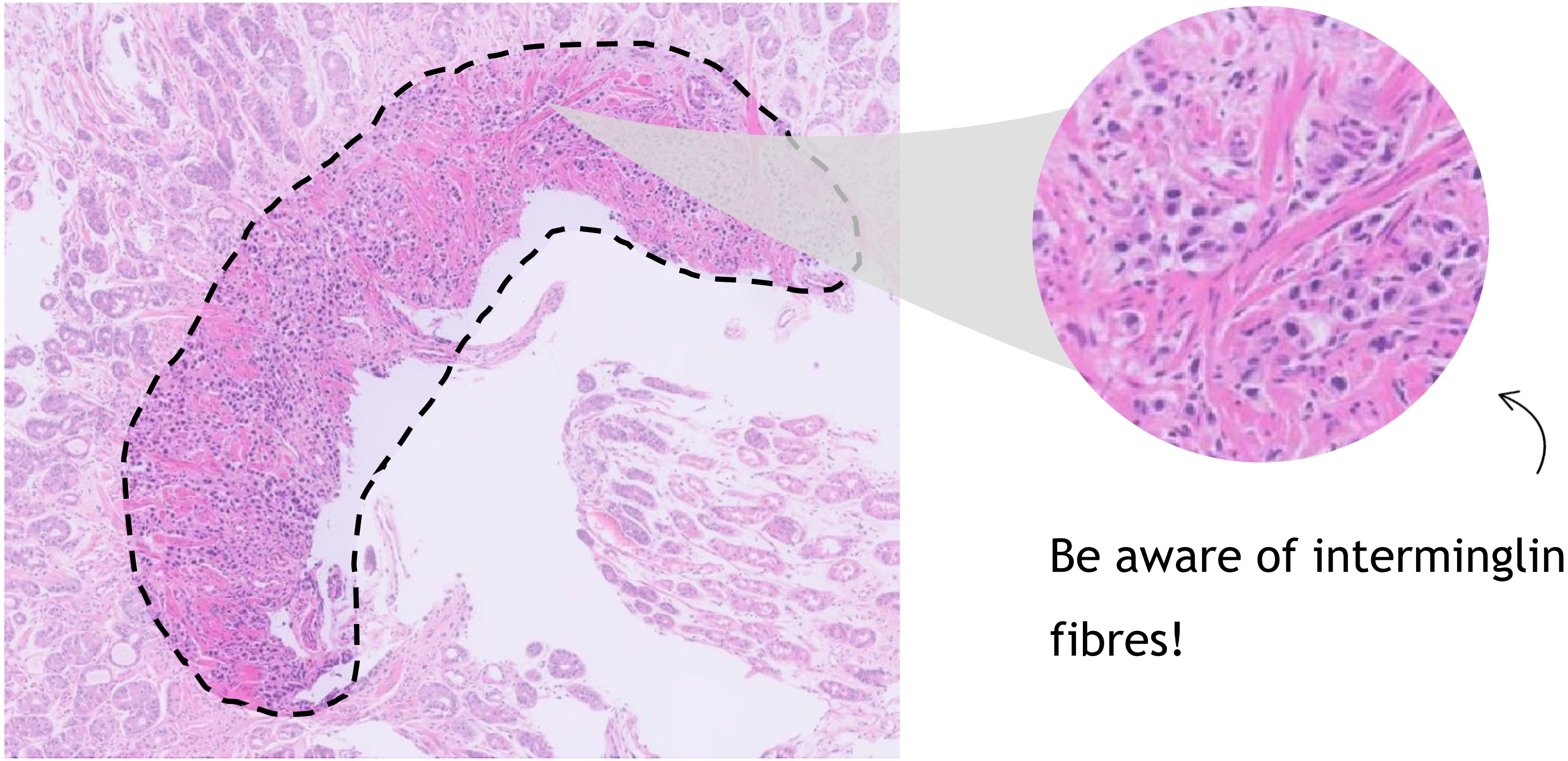

Be aware of intermingling muscle fibres!

Step 2. Define the stromal area

- Focus exclusively on stromal TILs
- Exclude extracellular mucin, intraluminal space of malignant glands and intratumoral vessels

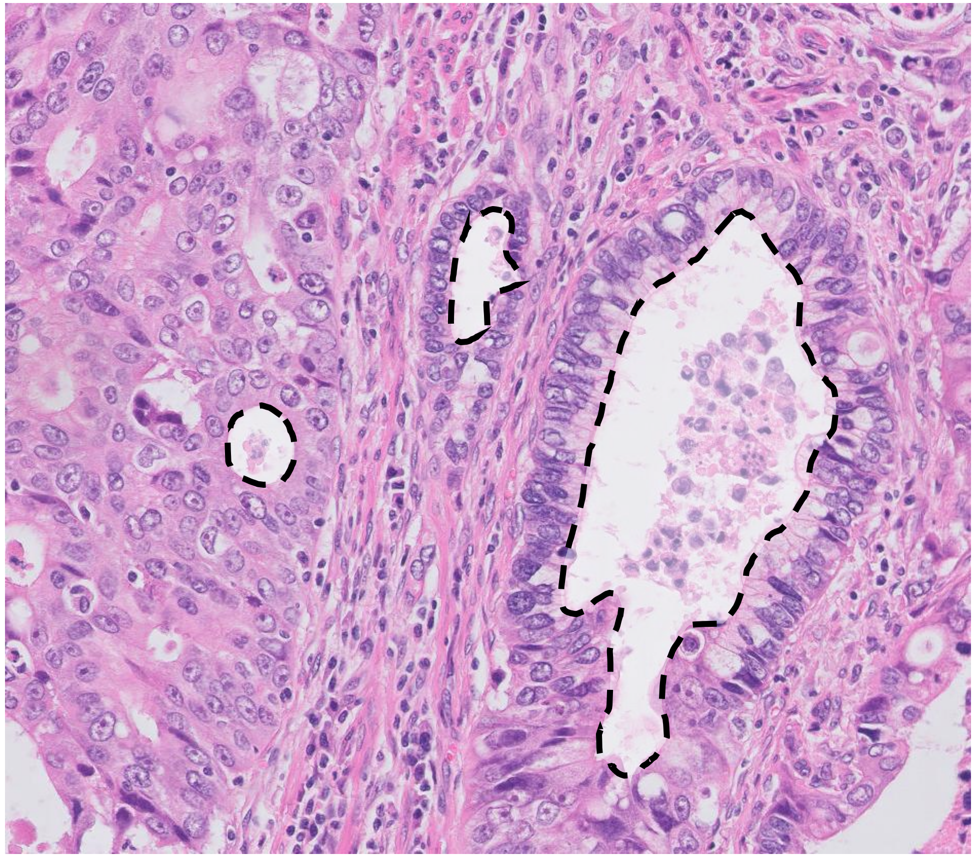

Step 2. Define the stromal area

- Focus exclusively on stromal TILs
- If tumor and stroma are indistinguishable, report TILs as a single total score without compartment distinction

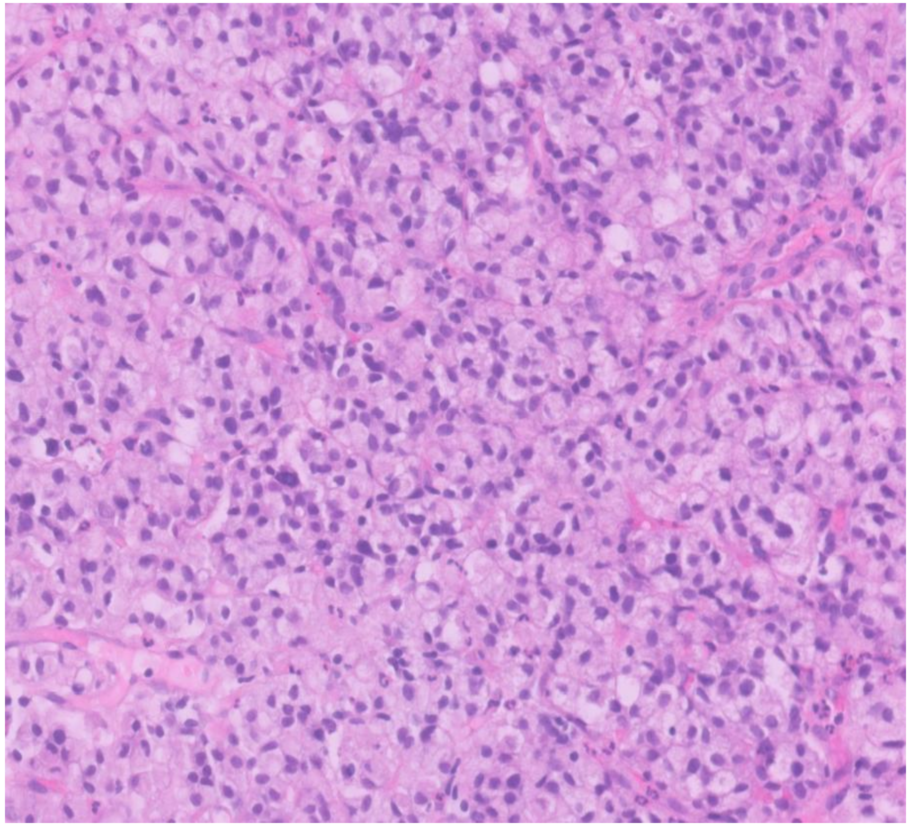

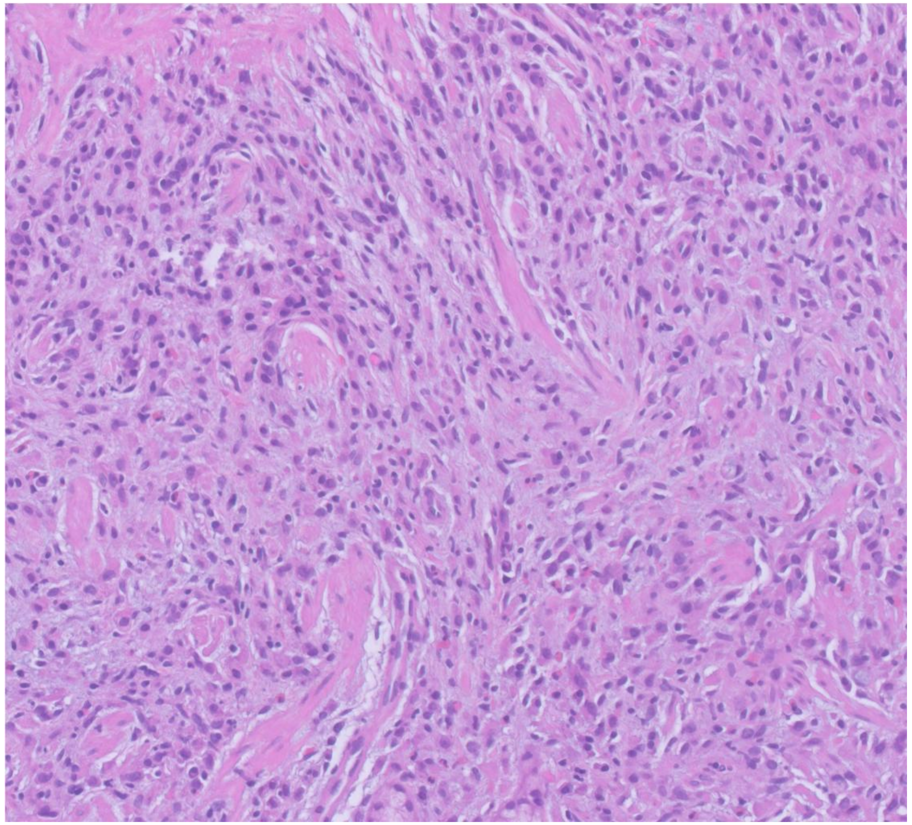

Step 3. Determine the type of immune infiltrate

- Include only mononuclear infiltrate (lymphocytes and plasma cells)
- Avoid the inclusion of granulocytes and other polymorphonuclear cells

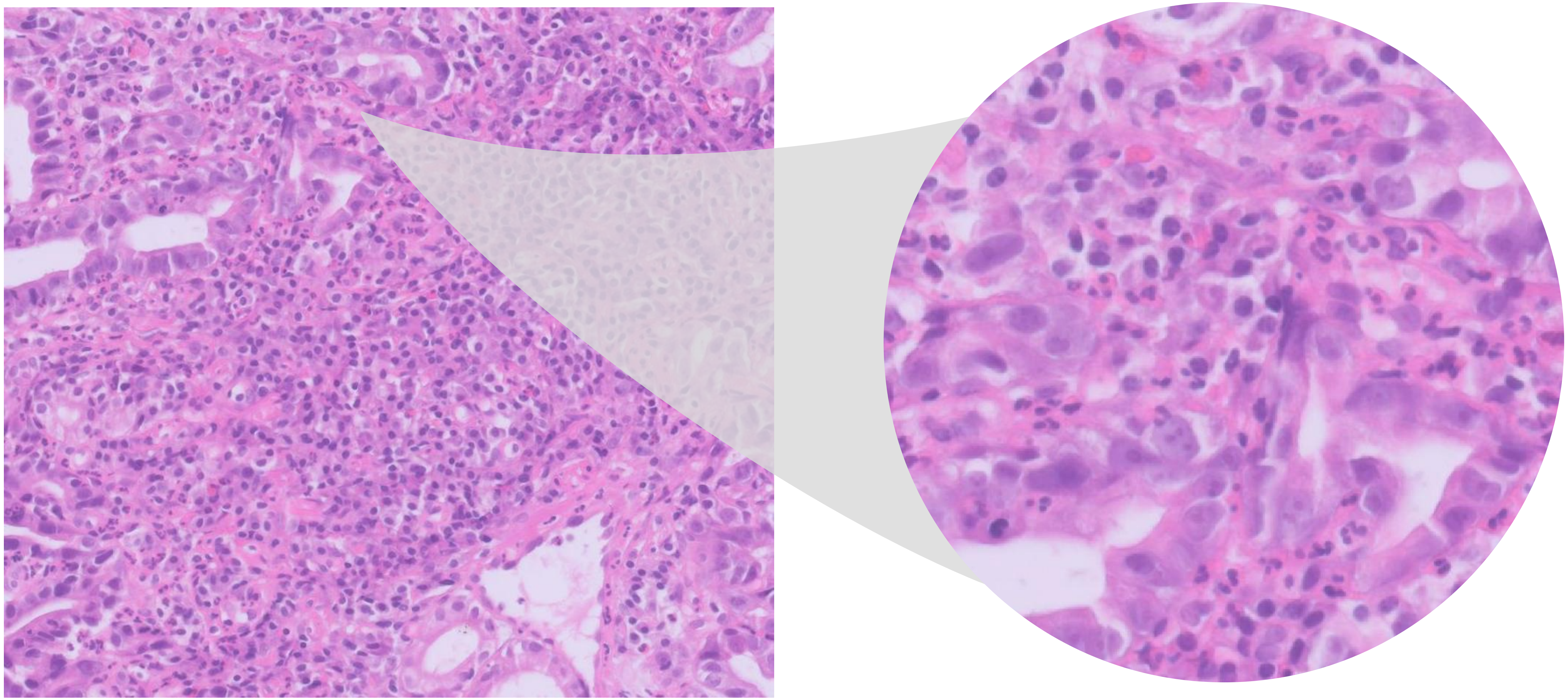

Step 3. Determine the type of immune infiltrate

- Include only mononuclear infiltrate (lymphocytes and plasma cells)
- Avoid the inclusion of granulocytes and other polymorphonuclear cells

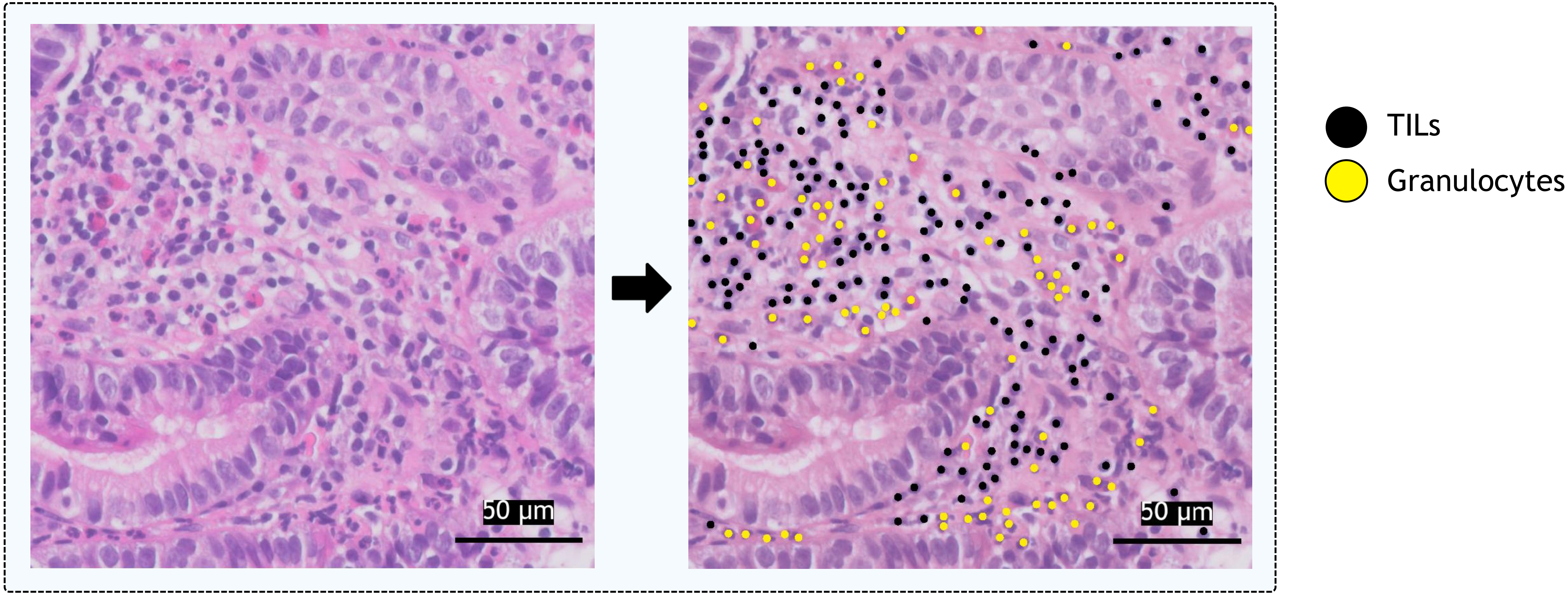

Step 4. Determine the percentage of stromal TILs

- Report the average of TILs in the stromal area, do not focus on hot spots.

$$\frac{\text{Total area TILs}}{\text{Total area tumor associated stroma}} * 100 = \textbf{TILs\%}$$

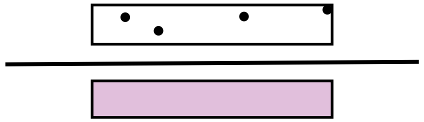

* 100 = **TILs%**

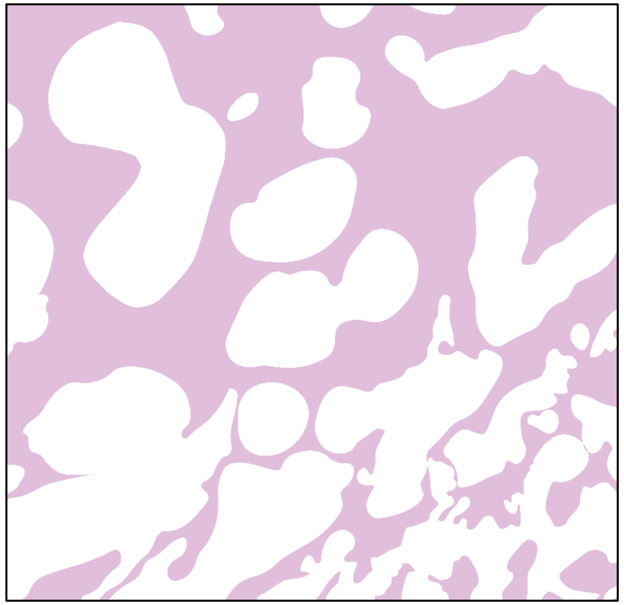

Tumor associated stroma

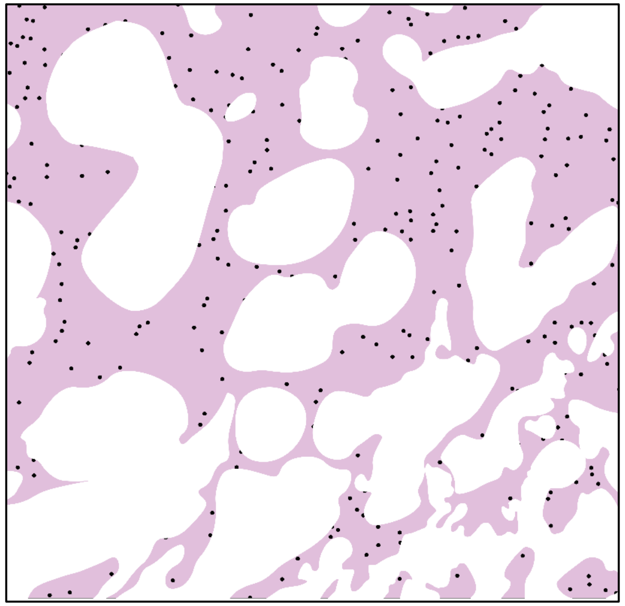

TILs

Step 4. Determine the percentage of stromal TILs

- Report the average of TILs in the stromal area, do not focus on hot spots.
- For intermediate groups evaluate different areas at higher magnification.

0-10% (low)	11-50% (intermediate)	51-90% (high)

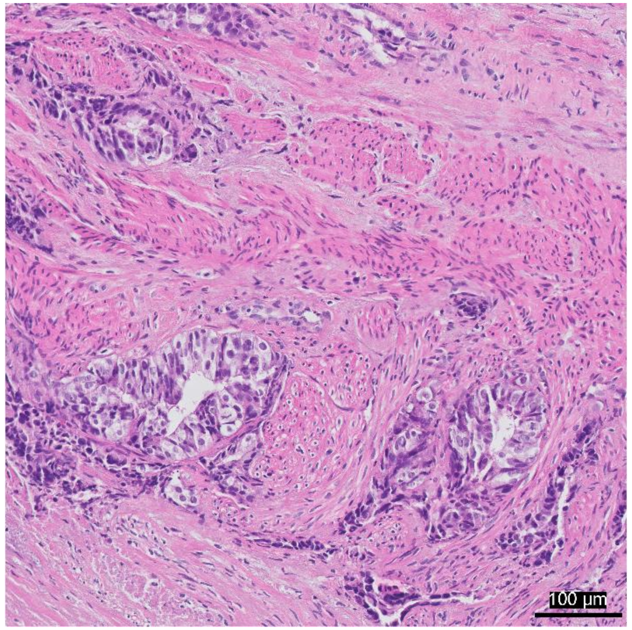

For this intermediate group evaluate different areas at higher magnification

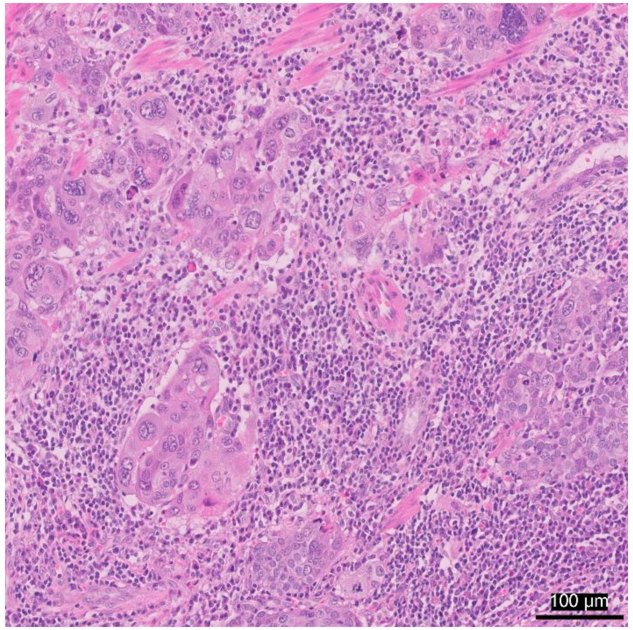

Step 4. Determine the percentage of TILs

- Report the average of TILs in the stromal area, do not focus on hot spots.
- For intermediate group evaluate different areas at higher magnification.
- Keep in mind that lymphocytes do not form solid aggregates, so even in cases with 90-100% stromal TILs, there will still be some space between the individual lymphocytes

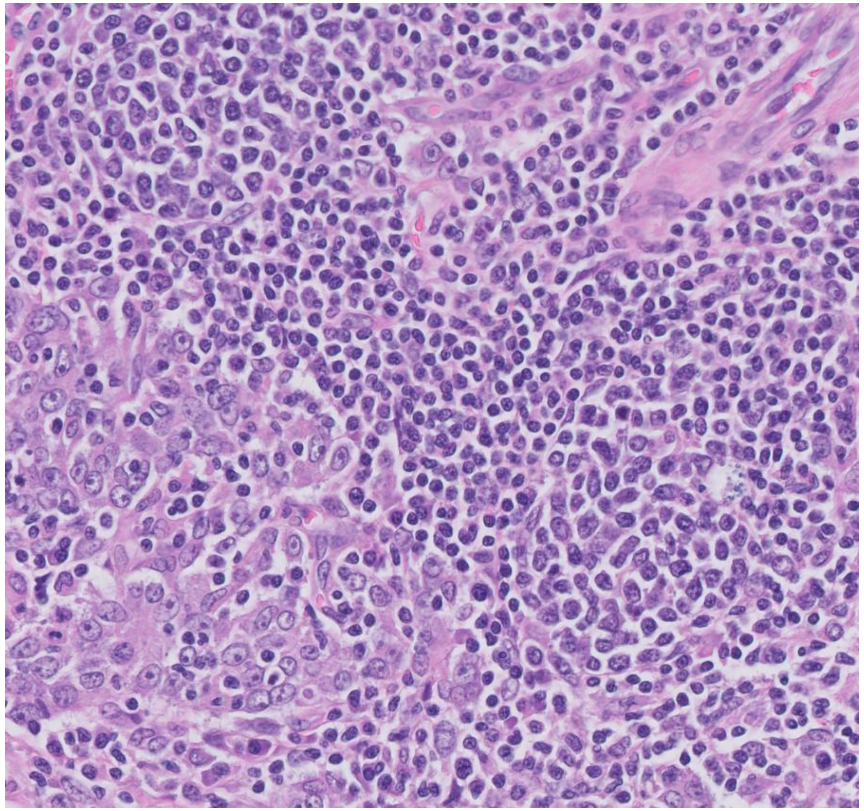

Reference images

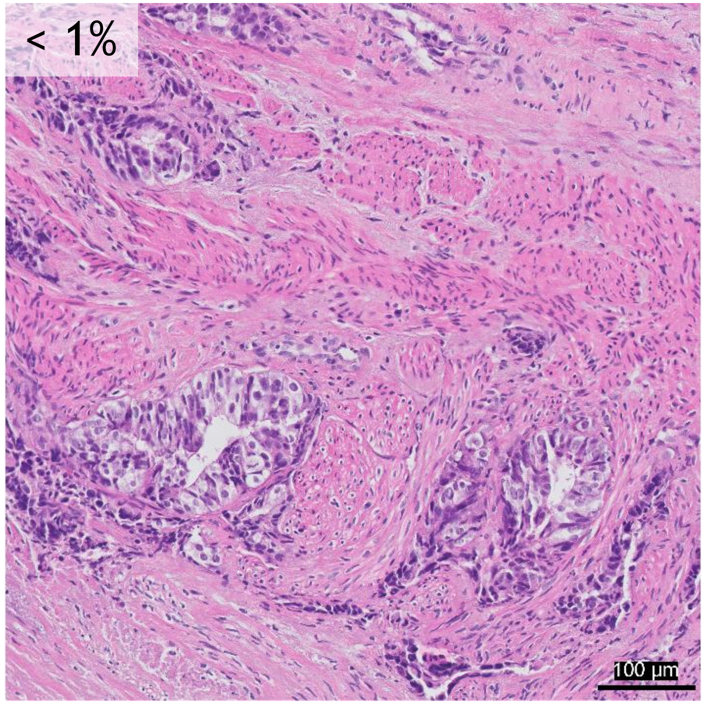

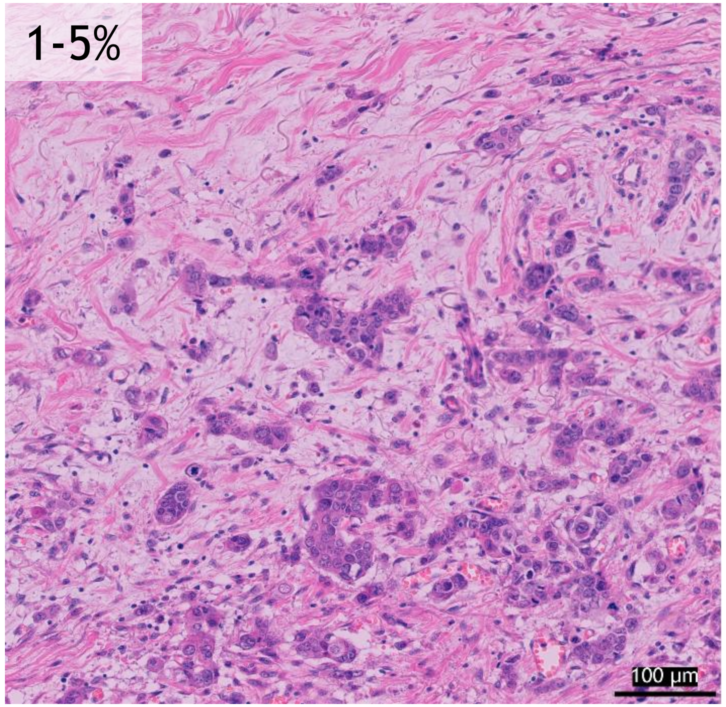

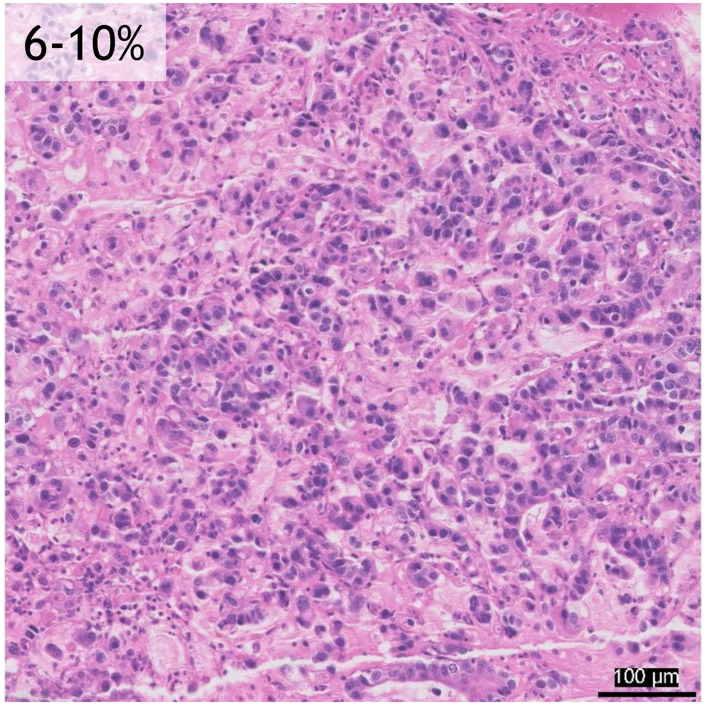

Reference images

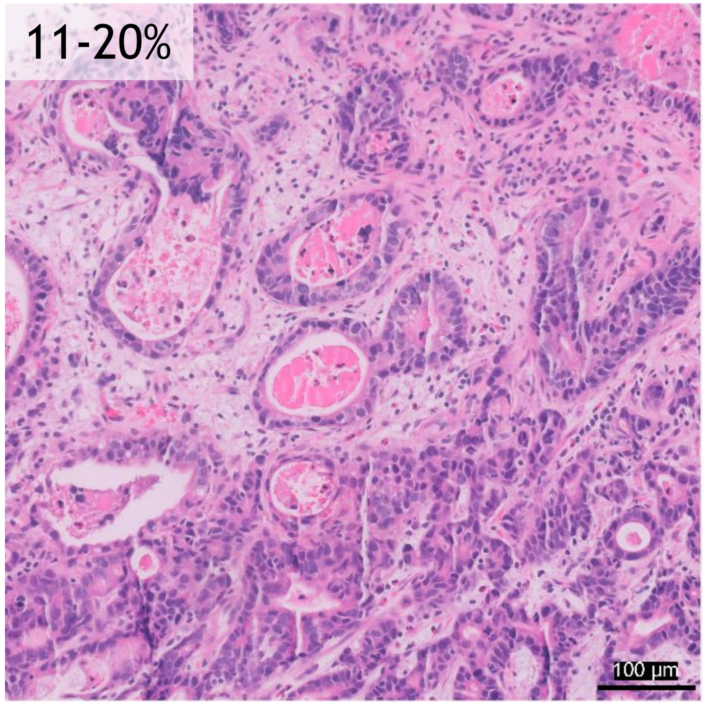

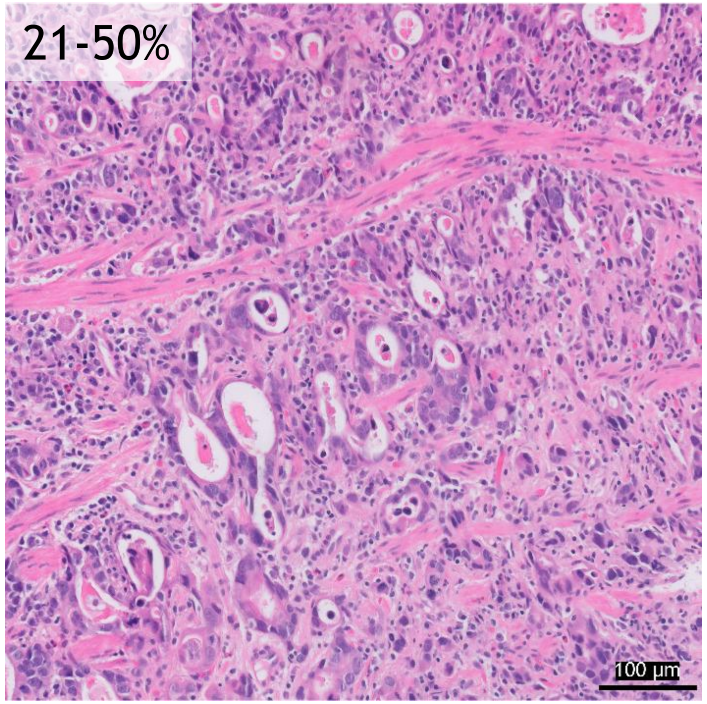

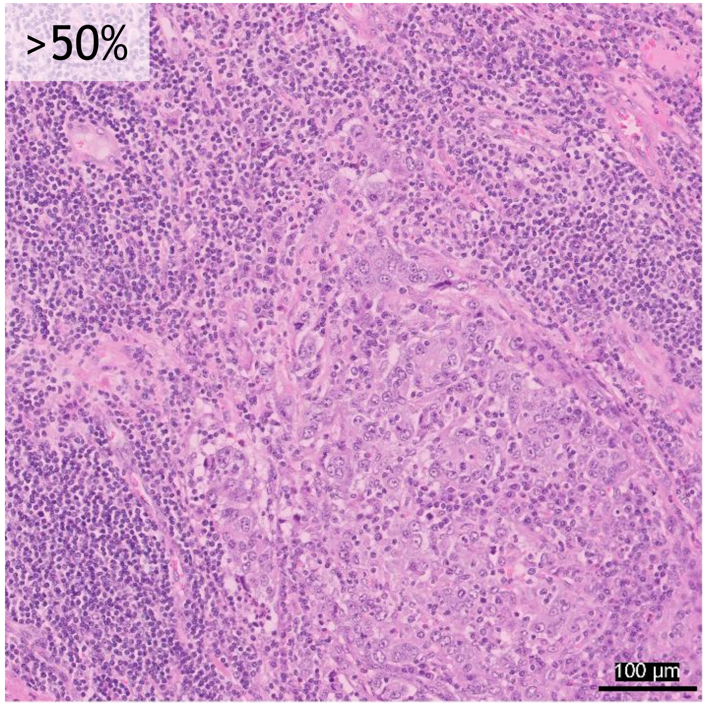

Schematic reference images

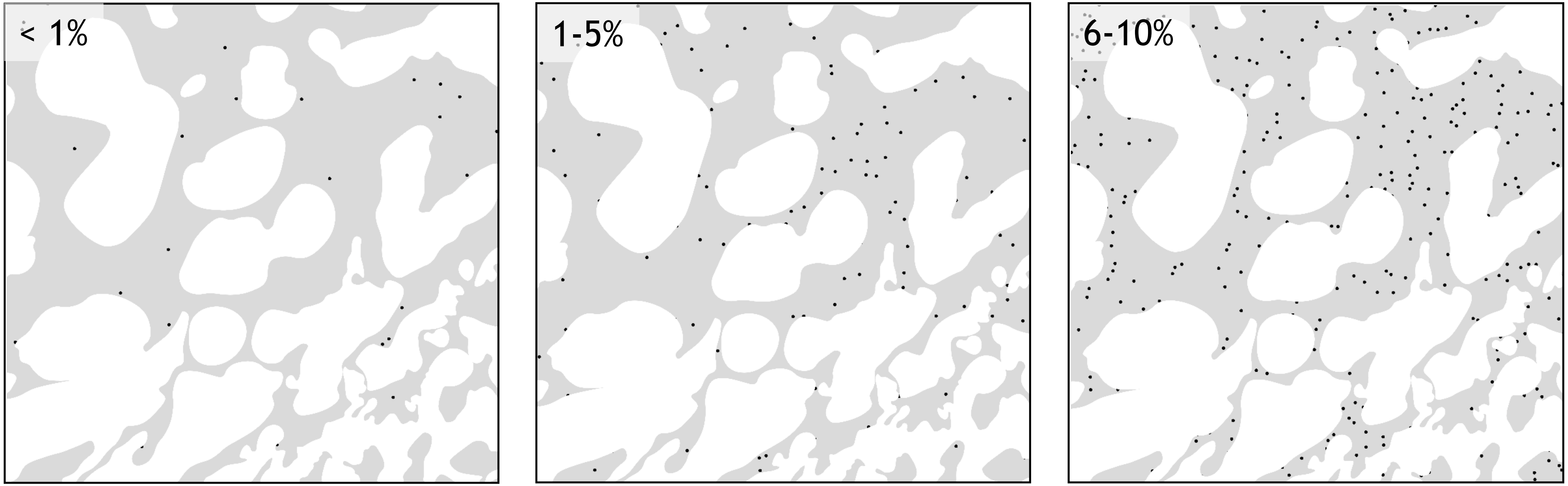

Schematic reference images

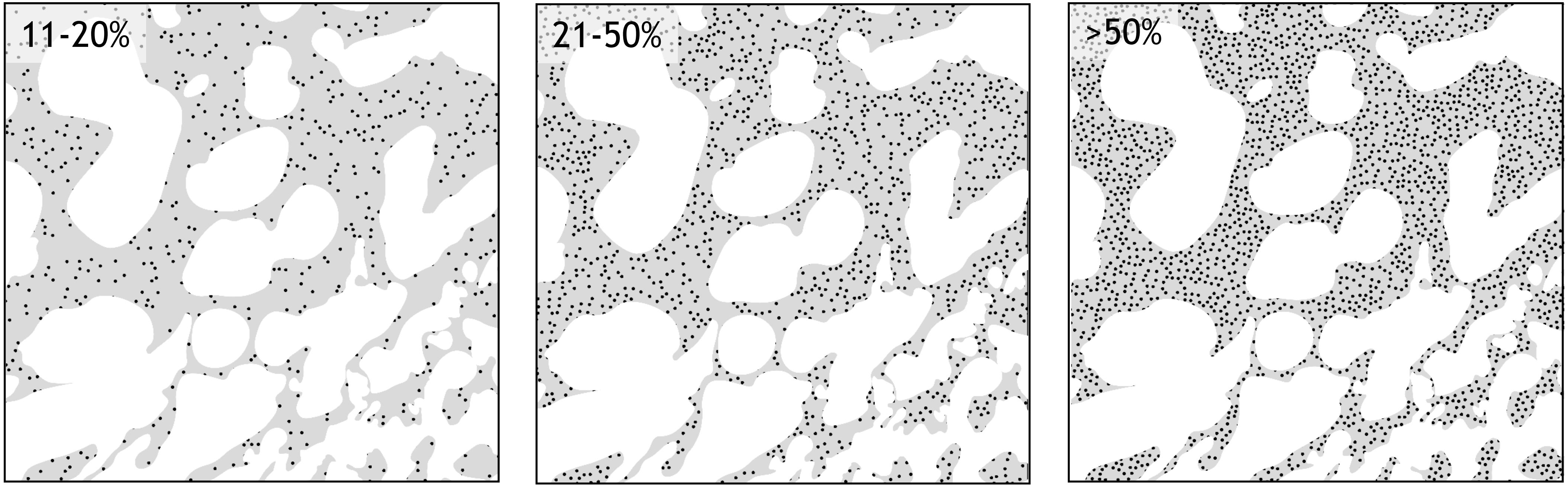

Supplement: Supplementary file 1 — Data S1. Upper_GI_tutorial. [file HIS-88-1126-s001.pdf]
