# Supplementary material for: Making sense of TILs: recommendations for morphological assessment of tumour‐infiltrating lymphocytes in gastro‐oesophageal carcinoma: A report on behalf of the International Immuno‐Oncology Biomarker Working Group
Source: Histopathology. 2026 Feb 5;88(6):1126–41. doi: 10.1111/his.70089 (PMC13051458; doi:10.1111/his.70089)
Supplement: Supplementary file 2 — Table S1. Proposed framework for TIL assessment in gastro‐oesophageal carcinoma specimens [file HIS-88-1126-s002.zip › his70089-sup-0001-TableS1-S1@Supplementary Materials.docx]

**Supplementary Materials**


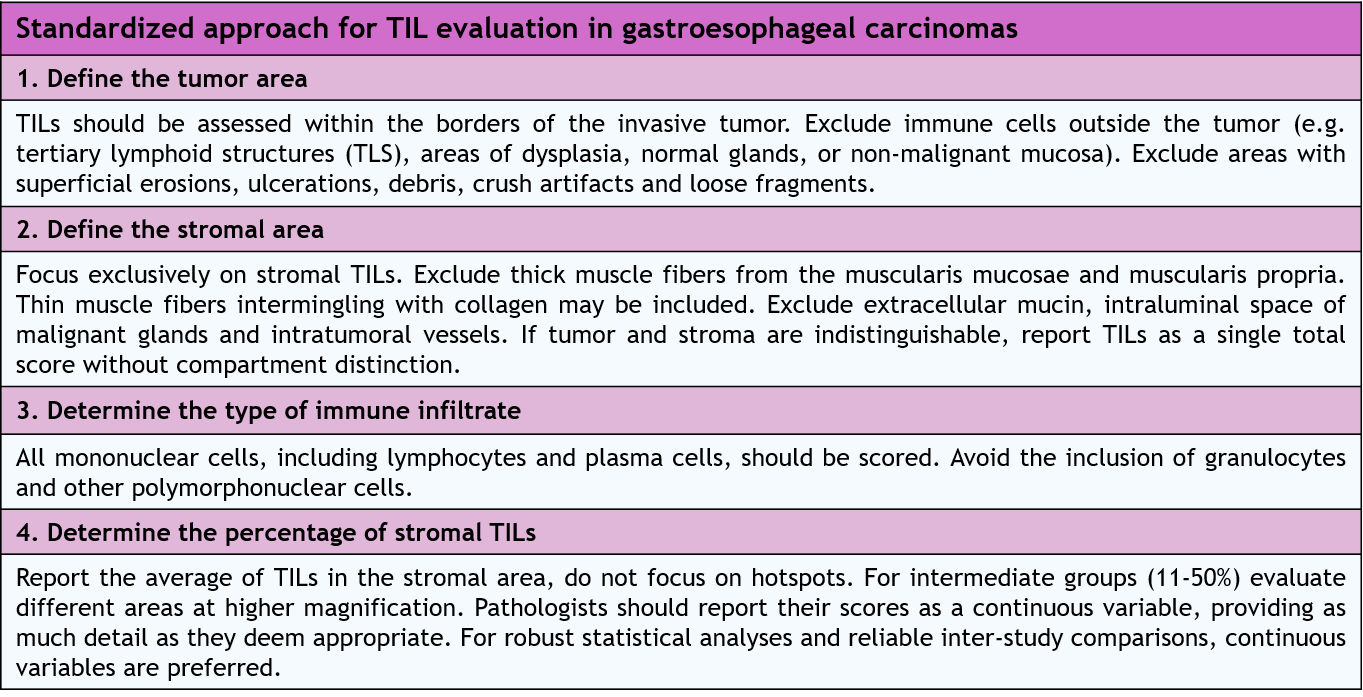


**Table S1**. Proposed framework for TIL assessment in gastroesophageal carcinoma specimens.
