# Supplementary material for: Making sense of TILs: recommendations for morphological assessment of tumour‐infiltrating lymphocytes in gastro‐oesophageal carcinoma: A report on behalf of the International Immuno‐Oncology Biomarker Working Group
Source: Histopathology. 2026 Feb 5;88(6):1126–41. doi: 10.1111/his.70089 (PMC13051458; doi:10.1111/his.70089)
Supplement: Supplementary file 2 — Table S1. Proposed framework for TIL assessment in gastro‐oesophageal carcinoma specimens [file HIS-88-1126-s002.zip › his70089-sup-0002-TableS1@Table S1.pdf]

Standardized approach for TIL evaluation in gastroesophageal carcinomas
**1. Define the tumor area**
TILs should be assessed within the borders of the invasive tumor. Exclude immune cells outside the tumor (e.g. tertiary lymphoid structures (TLS), areas of dysplasia, normal glands, or non-malignant mucosa). Exclude areas with superficial erosions, ulcerations, debris, crush artifacts and loose fragments.
**2. Define the stromal area**
Focus exclusively on stromal TILs. Exclude thick muscle fibers from the muscularis mucosae and muscularis propria. Thin muscle fibers intermingling with collagen may be included. Exclude extracellular mucin, intraluminal space of malignant glands and intratumoral blood vessels. If tumor and stroma are indistinguishable, report TILs as a single total score without compartment distinction.
**3. Determine the type of immune infiltrate**
All mononuclear cells, including lymphocytes and plasma cells, should be scored. Avoid the inclusion of granulocytes and other polymorphonuclear cells.
**4. Determine the percentage of stromal TILs**
Report the average of TILs in the stromal area, do not focus on hotspots. For intermediate groups (11-50%) evaluate different areas at higher magnification. Pathologists should report their scores as a continuous variable, providing as much detail as they deem appropriate. For robust statistical analyses and reliable inter-study comparisons, continuous variables are preferred.
